# Supplementary figures and images for: Overlaid positive and negative feedback loops shape dynamical properties of PhoPQ two-component system
Source: PLoS Comput Biol. 2021 Jan 4;17(1):e1008130. doi: 10.1371/journal.pcbi.1008130 (PMC7808668; doi:10.1371/journal.pcbi.1008130)

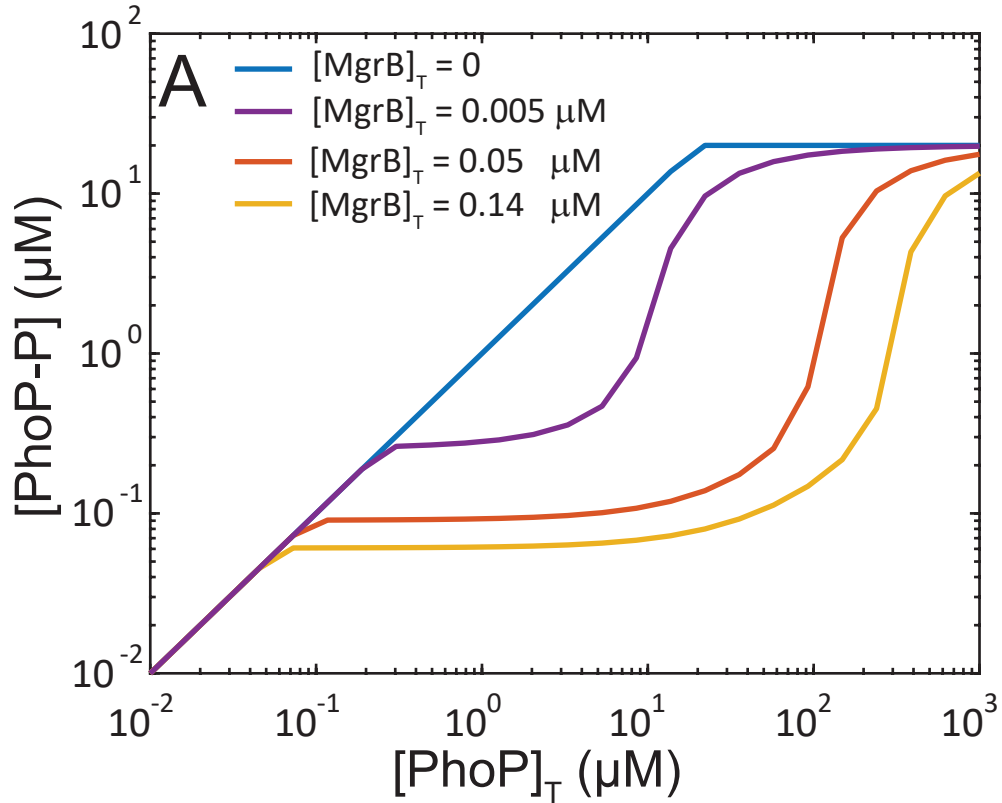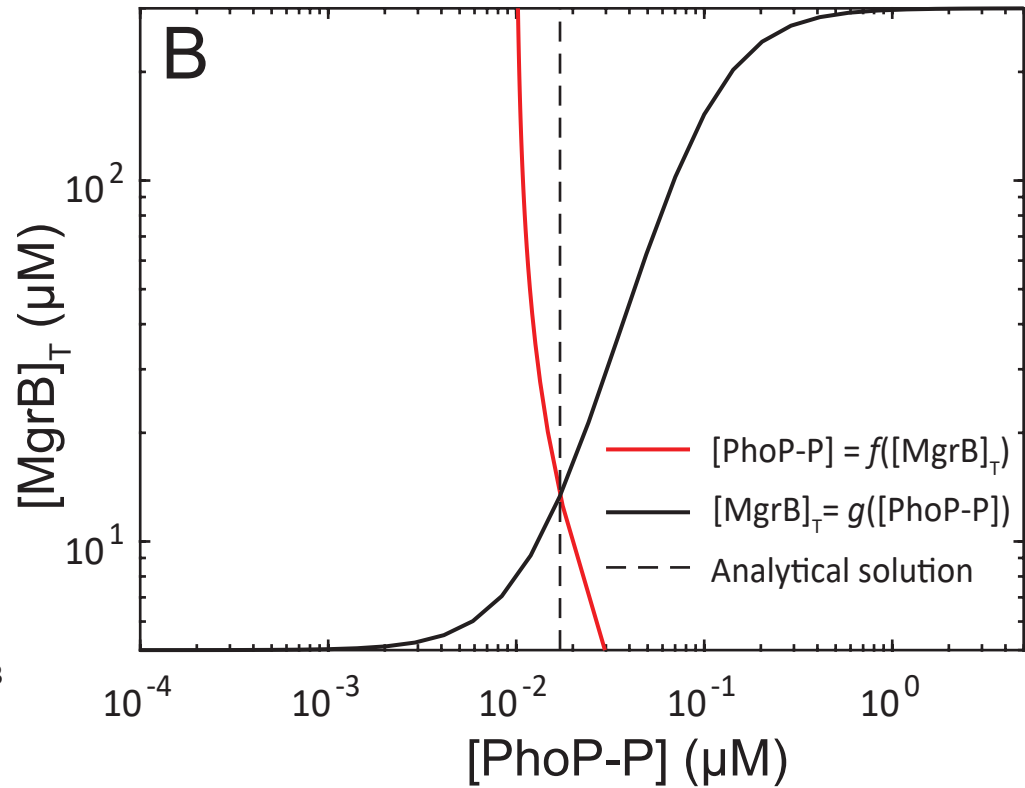

Supplement: S1 Fig — Steady state [PhoP-P] as a function of total PhoP, PhoQ at various [MgrB]T0 levels (all concentrations in units of (μM). As [MgrB]T0 increases, a range of total-PhoP,PhoQ appears where PhoP-P is robust (B) Each point in (A) is an intersection of transcription and interaction modules (Fig 2A, main text). Red curve shows solution to the interaction module. At fixed total PhoP,PhoQ, total-MgrB is increased and steady state [PhoP-P] is computed. Black curve represents the saturating dependence of MgrB-total on [PhoP-P]. The dotted line is the analytical solution obtained by solving Eqs 1 and 2 in main text. (PDF) [file pcbi.1008130.s001.pdf]

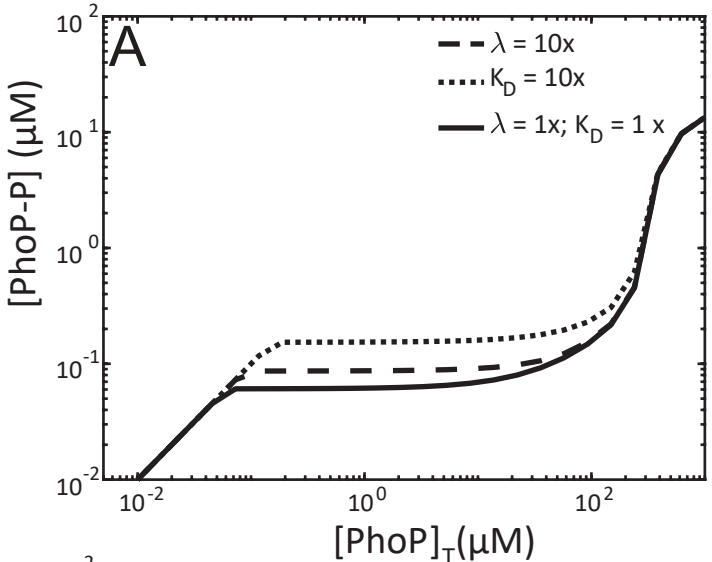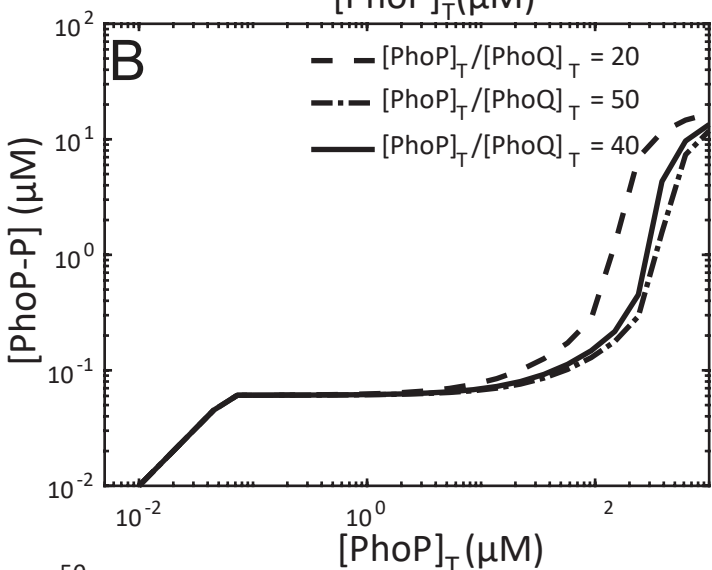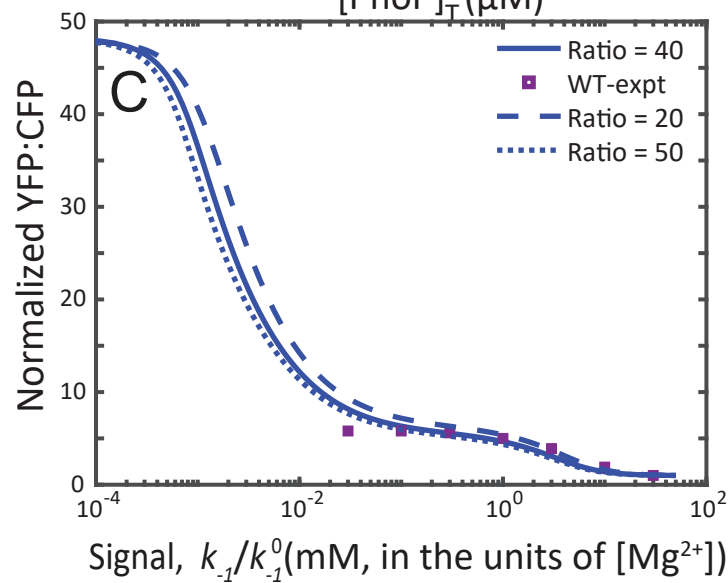

Supplement: S2 Fig — (A,B) Phenomenon of robustness to PhoPQ variation is observed in the one-state PhoPQ model over varying λ, KD values or over varying ratio of PhoP:PhoQ. (C) Variations in PhoP:PhoQ ratio also do not change the conclusions of biphasic steady-state response of the two-state model. (PDF) [file pcbi.1008130.s002.pdf]

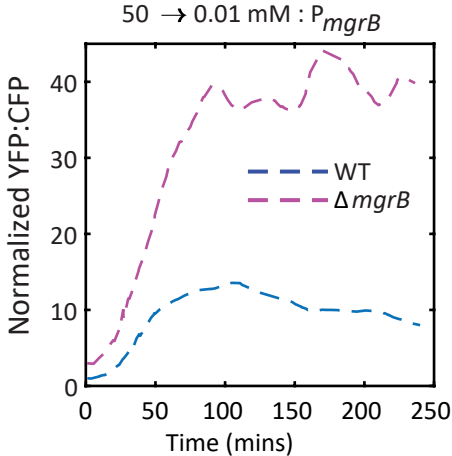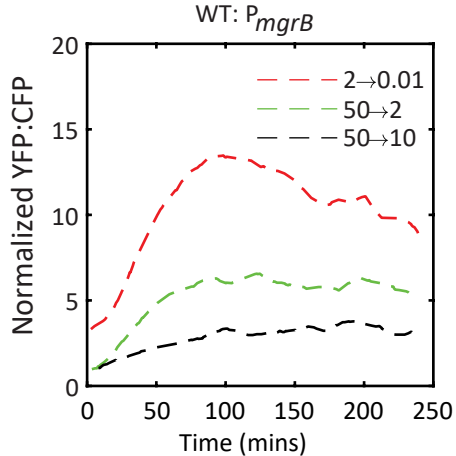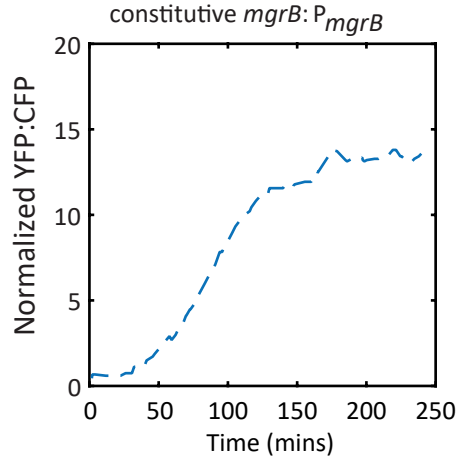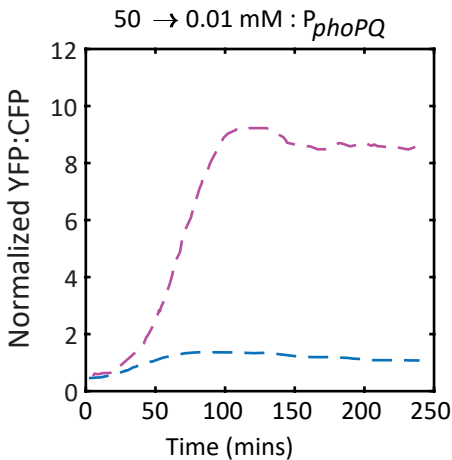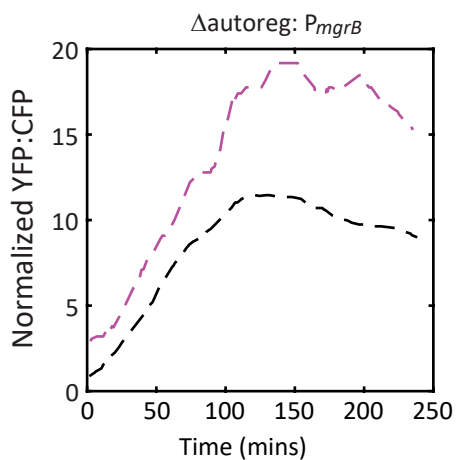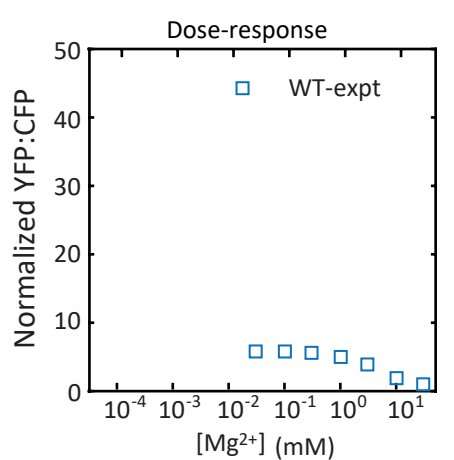

Supplement: S3 Fig — YFP:CFP ratio (normalized to the ratio at 50mM) extracted from [16] and [21]. This normalized data set was used to fit temporal and steady state parameters for all models described in this paper. Data was extracted using image analysis in MATLAB (except bottom right panel, which was read out manually from ref [21]). All data except constitutive mgrB (top right panel) was used to fit models. (PDF) [file pcbi.1008130.s003.pdf]

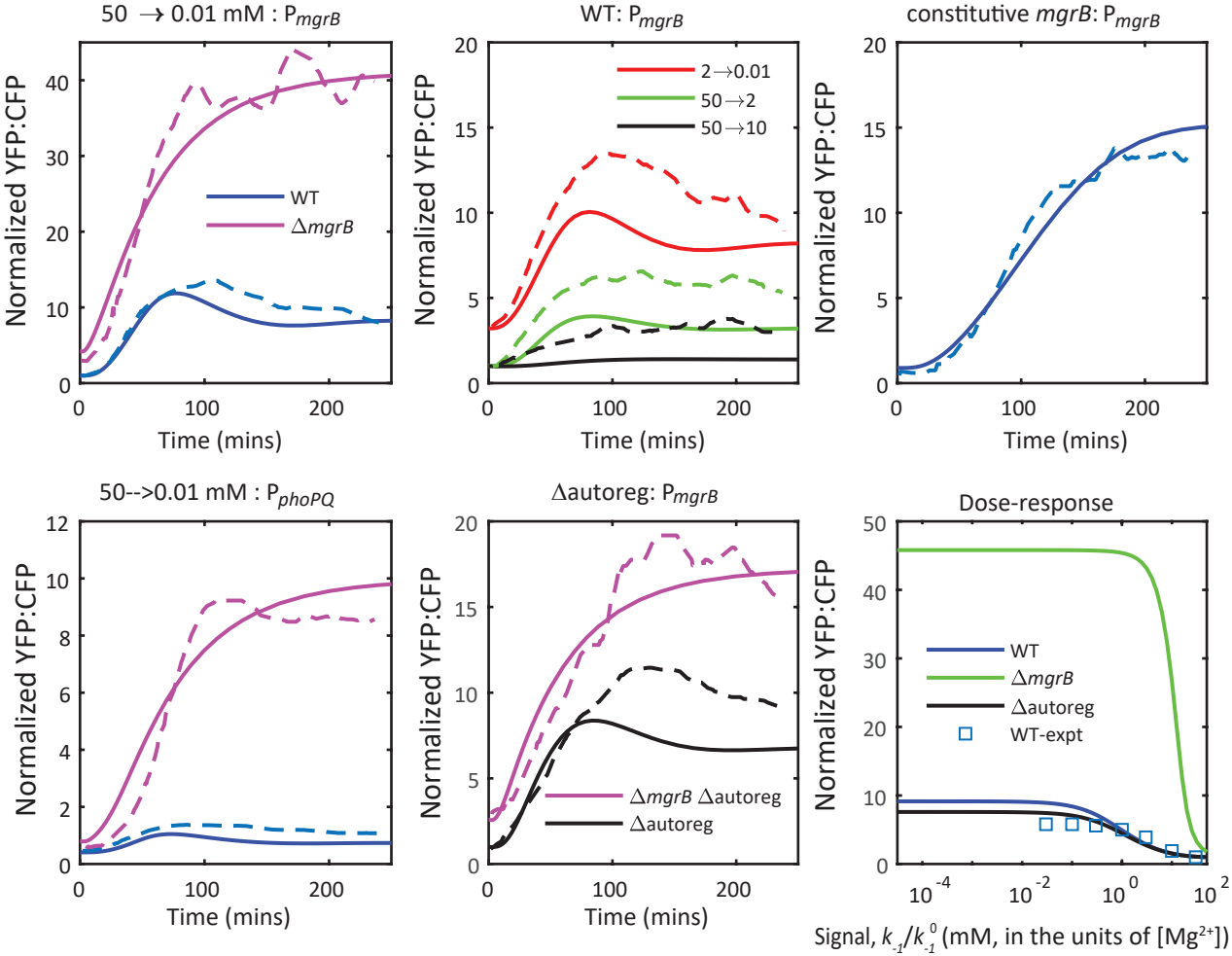

Supplement: S4 Fig — Simulations from a representative parameter set showing best quantitative fit for the simple PhoPQ model. Simulation of an in-silico mutant expressing mgrB constitutively (top right, solid line) at 10x basal transcription rate of mgrB in wild-type. This simulation in addition to PhoQ phosphatase-lacking mutant was used to verify whether a parameter set was accurate. (PDF) [file pcbi.1008130.s004.pdf]

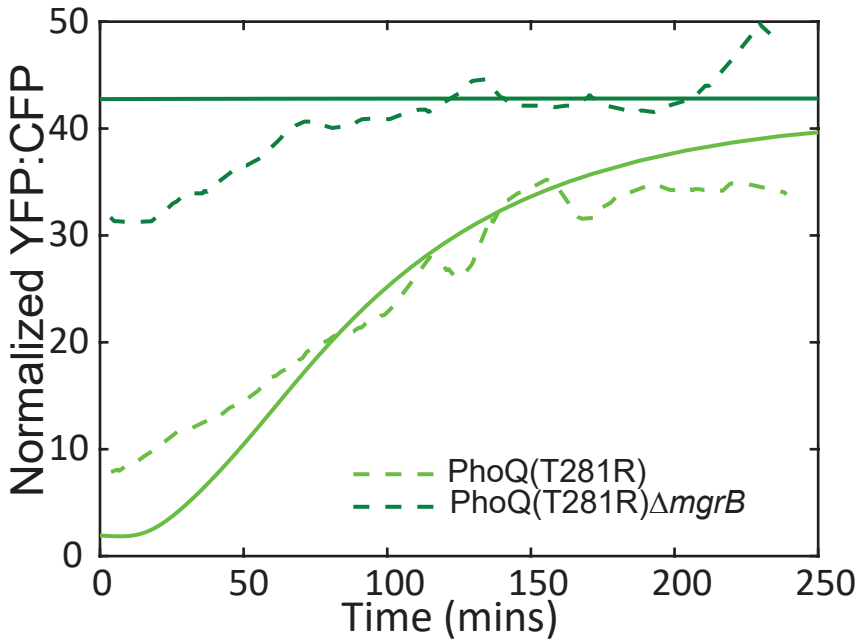

Supplement: S5 Fig — With parameters that generate the fit in S6 Fig, we simulate the Mg2+ step-down with an in-silico mutant of PhoQ lacking phosphatase activity single mutant (light green, solid line) or double mutant with mgrB-deletion (dark green, solid line). These simulations show a qualitative match with the experimental data (dashed lines). (PDF) [file pcbi.1008130.s005.pdf]

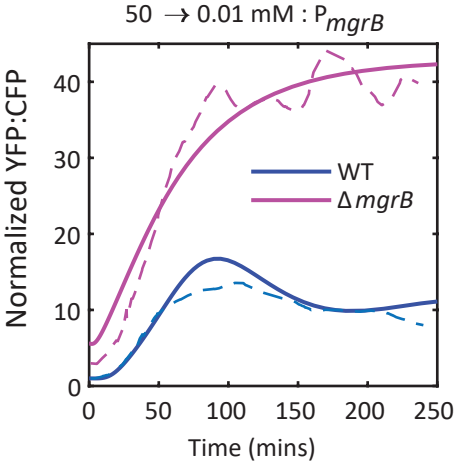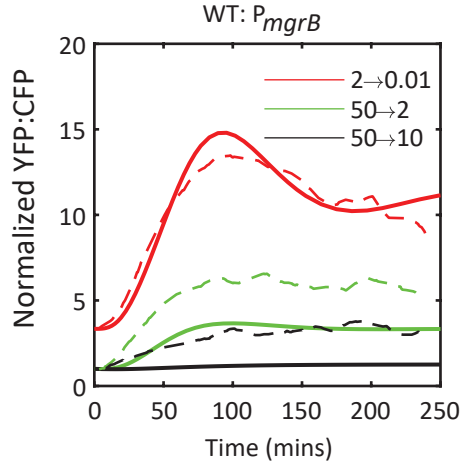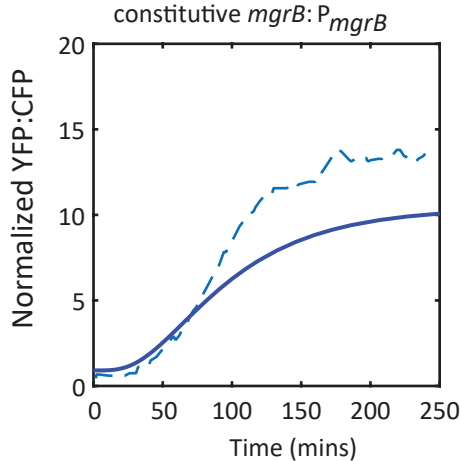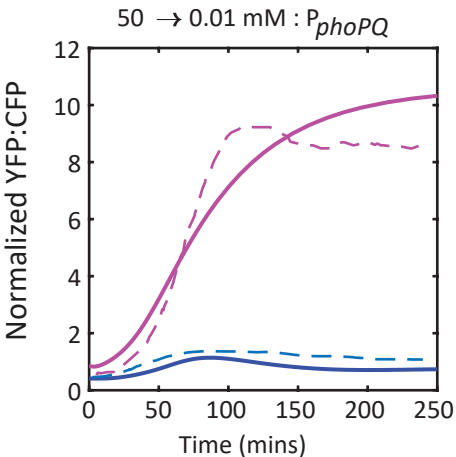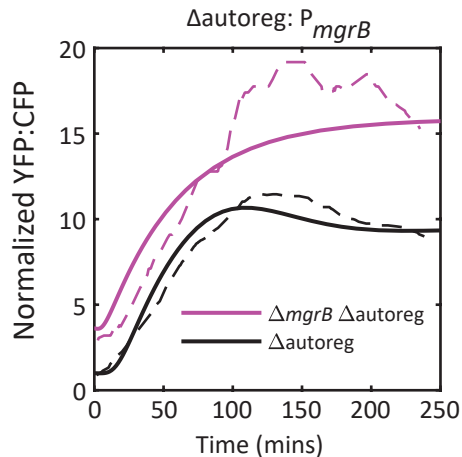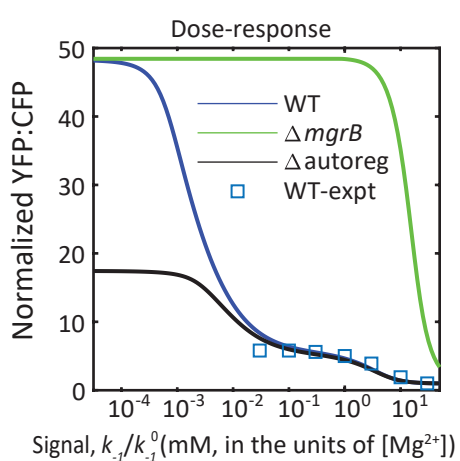

Supplement: S6 Fig — Simulations from a representative parameter set showing best quantitative fit for the two-state PhoPQ model. (PDF) [file pcbi.1008130.s006.pdf]

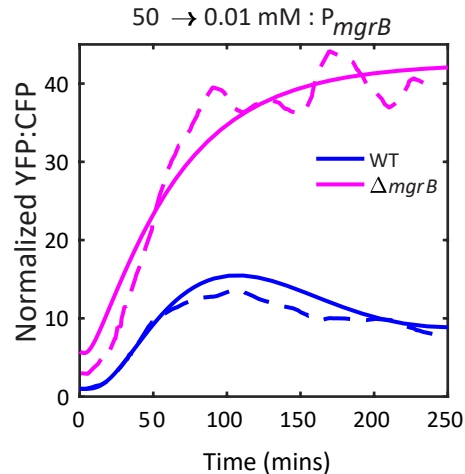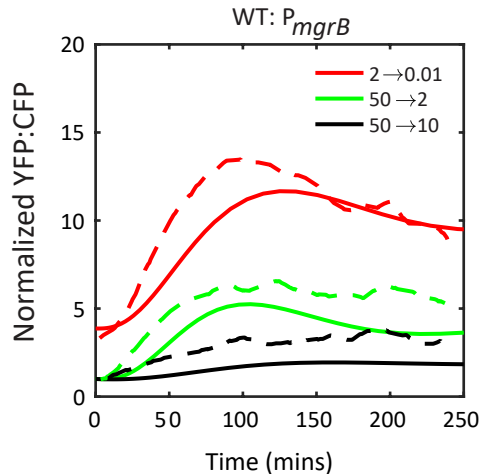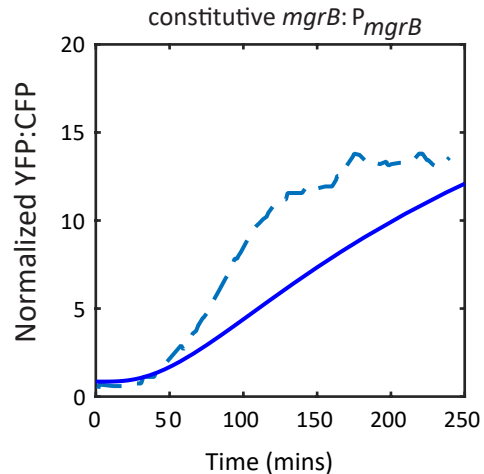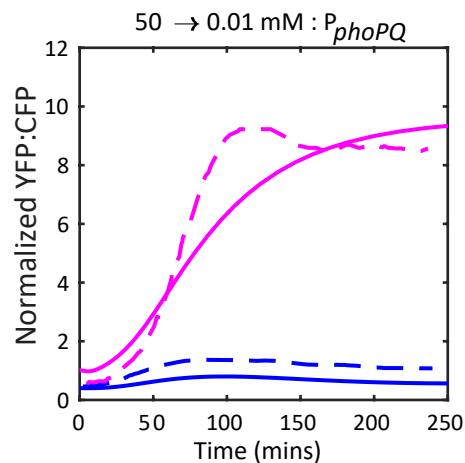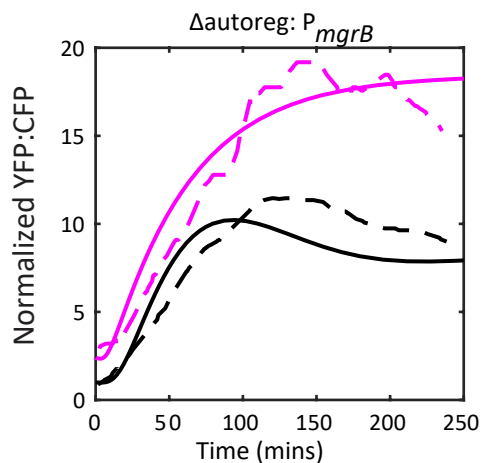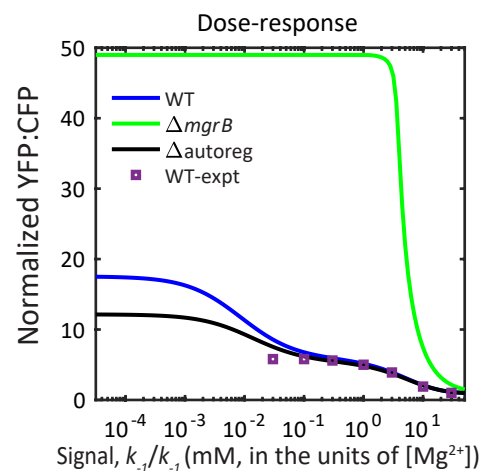

Supplement: S7 Fig — Simulations from a representative parameter set showing best quantitative fit for the two-state PhoPQ model with lower maximum output relative to plateau level. (PDF) [file pcbi.1008130.s007.pdf]

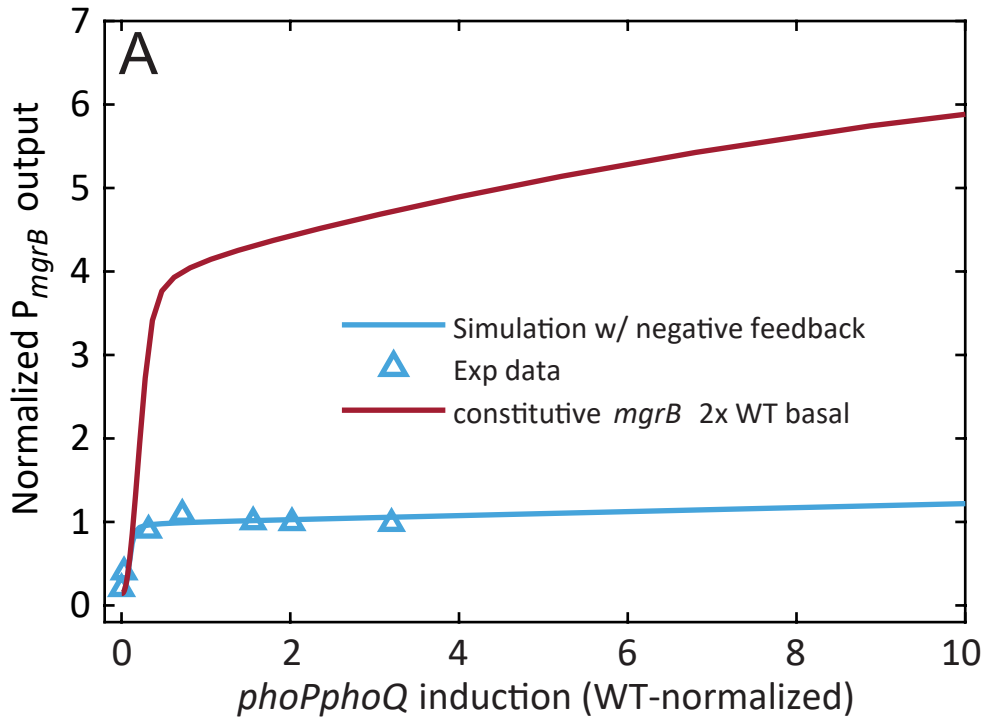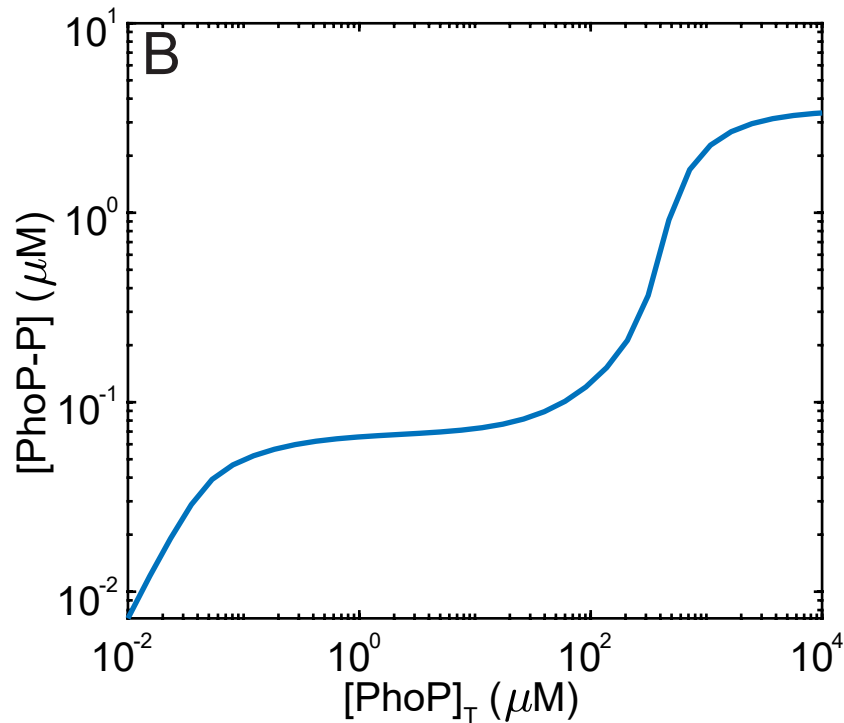

Supplement: S8 Fig — (A) Blue line shows simulated steady state expression from the PmgrB promoter at 1mM Mg2+ from an in silico mutant with PphoPQ promoter under inducible control (instead of autoregulated). PhoPQ TCS model also predicts that PhoP-P output will not be robust to overexpression of PhoP and PhoQ if mgrB is expressed constitutively instead of under PhoP-P control (red line). Promoter expression is shown as YFP:CFP normalized to steady state WT YFP:CFP ratio at the same Mg2+. The x-axis shows total [PhoP] from the in silico mutant simulation normalized to WT total [PhoP]. The axes are recreated from Fig 4C in [21], and blue triangles represent data from the same figure. (B) Over a larger range of total-PhoP, the two-state model also shows a range of PhoP expression in which PhoP-P does not vary significantly similar to one-state model. (PDF) [file pcbi.1008130.s008.pdf]
