# Supplementary material for: Overlaid positive and negative feedback loops shape dynamical properties of PhoPQ two-component system
Source: PLoS Comput Biol. 2021 Jan 4;17(1):e1008130. doi: 10.1371/journal.pcbi.1008130 (PMC7808668; doi:10.1371/journal.pcbi.1008130)
Supplement: S1 Text — (PDF) [file pcbi.1008130.s009.pdf]

S1 Text: Analyzing PhoPQ-MgrB reaction network for absolute concentration robustness

Note that for brevity in the figures and equations in all supplemental text files: We represent PhoQ by Q, MgrB by B and subscript P for phosphorylated state. For example, the complex PhoQ-P.MgrB is represented as QB_P.

Biochemical reaction networks can possess a property of absolute concentration robustness (ACR) based on the structure of the network alone [1]. If a biochemcial reaction network satisfies certain conditions related to the structure of the network, ACR can result in the concentration of one species being robust to variation in all other species concentrations. Here we analyze whether the interactions in PhoPQ TCS satisfy the conditions for ACR.

The biochemical reaction networks shown in Figure 1 are analyzed for properties such as nodes, strong linkage classes and rank. We follow the same graphical representation as Shinar and Feinberg. Strong linkage classes are enclosed in dotted outlines and linkage classes within solid outlines. We also color nodes by pink (terminal) or blue (non-terminal). The theorem given in ref [1] was used to identify absolute concentration robustness.

As shown in Figure 1A, in the case of a reversible autophosphorylation reaction, the nodes Q and Q_P are part of a single strong linkage class. As neither of the nodes of this strong linkage class reacts to any other strong linkage class, it is a terminal strong linkage class. Therefore both Q and Q_P are terminal nodes. As such, even though the deficiency of the network is 1, there is no pair of non-terminal nodes that differ in P_P. Therefore, in case of a reversible autophosphorlyation reaction, our network does not possess ACR.

On the other hand, in the limit of an irreversible autophosphorylation reaction (Figure 1B), the nodes Q and Q_P are each a separate strong linkage class. Given that the node Q reacts to Q_P, another strong linkage class, it is not a terminal strong linkage class. Therefore, Q is a non-terminal node. Together with the unit deficiency of the network, $(Q, Q + P_P)$ is a pair of non-terminal nodes differing in the species P_P, and the network possess ACR.

Regardless of the reversibility of autophosphorylation reaction, the reaction network featuring MgrB (Figure 1C) has a deficiency of 3. Thus, the conditions for ACR in the network are not met even with irreversible

autophosphorylation. The number of nodes, number of linkage classes and rank of the reaction network with or without MgrB is tabulated in Table 1.

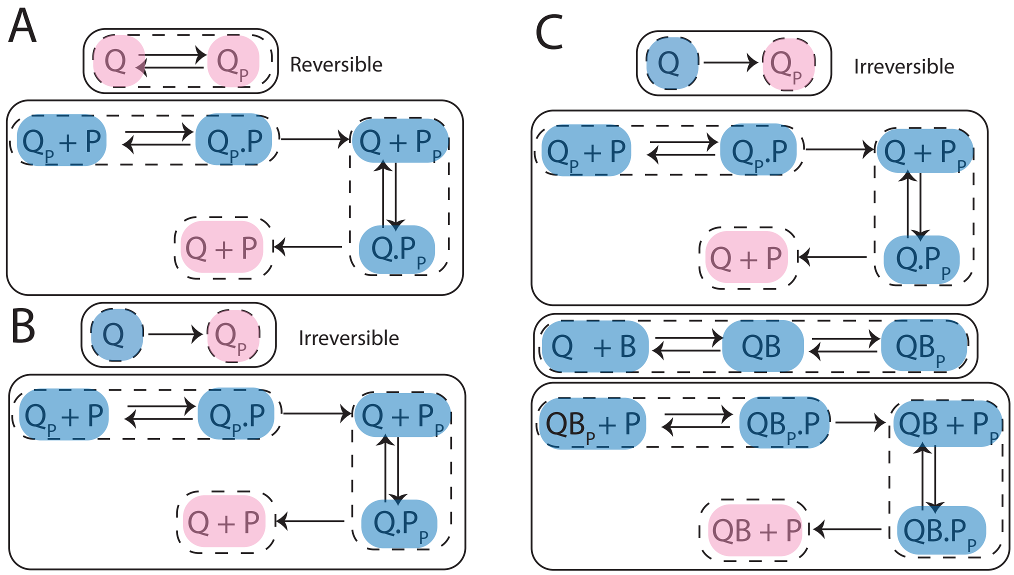

Figure 1: Biochemical reaction network for PhoPQ TCS is shown in A and B without MgrB and in C with MgrB. Dashed outlines indicate strong linkage classes, while solid outlines indicate linkage classes. Terminal nodes are colored pink while non-terminal nodes are colored blue. The autophosphorylation reaction is considered reversible (A) or irreversible (B).

Property	With MgrB	Without MgrB
Nodes (n)	15	7
Linkage classes (l)	4	2
Rank (r)	8	4
Deficiency $(n - l - r)$	3	1
Pair of non-terminal nodes differing in $\mathrm{P_P}$	NA	$(\mathrm{Q}, \mathrm{Q} + \mathrm{P_P})$
Satisfies condition for ACR ([1])	No	Yes

Table 1: Network-analysis of PhoPQ TCS interactions

References

[1] G. Shinar and M. Feinberg. Structural sources of robustness in biochemical reaction networks. *Science*, 327(5971):1389–91, 2010.
