# Supplementary material for: Overlaid positive and negative feedback loops shape dynamical properties of PhoPQ two-component system
Source: PLoS Comput Biol. 2021 Jan 4;17(1):e1008130. doi: 10.1371/journal.pcbi.1008130 (PMC7808668; doi:10.1371/journal.pcbi.1008130)
Supplement: S2 Text — (PDF) [file pcbi.1008130.s010.pdf]

S2 Text: Steady state PhoP-P for one-state PhoPQ-MgrB model

Note that for brevity in the figures and equations in all supplemental text files: We represent PhoQ by Q, MgrB by B and subscript P for phosphorylated state. For example, the complex PhoQ-P.MgrB is represented as $\mathrm{QB_P}$.

In this section we identify conditions under which PhoP-P is robust to overexpression of PhoP and PhoQ (Figure 2, Main text). We find an approximate analytical solution to this model that is the simplest modification of the PhoPQ model created by Miyashiro and Goulian in [3]. Reactions and ODEs are described in S5 Text. Phosphotransfer and phosphatase complexes at steady state give:

$$\begin{aligned}
K_{MT} &= \frac{[\mathrm{Q_P}][\mathrm{P}]}{[\mathrm{Q_P.P}]} \\
K_{MTb} &= \frac{[\mathrm{QB_P}][\mathrm{P}]}{[\mathrm{QB_P.P}]} \\
K_{MP} &= \frac{[\mathrm{Q}][\mathrm{P_P}]}{[\mathrm{Q.P_P}]} \\
K_{MPb} &= \frac{[\mathrm{QB}][\mathrm{P_P}]}{[\mathrm{QB.P_P}]}
\end{aligned} \tag{1}$$

K_{MP}, K_{MPb},K_{MT},K_{MTb} are Michaelis-Menten constants for the catalytical reactions shown in Figure 1 S5 Text. At steady state, the flux of phosphorylation of PhoP can be matched with the flux of dephosphorylation of PhoP-P. This gives

$$k_t[\mathrm{Q_P.P}] + k_{tb}[\mathrm{QB_P.P}] = k_p[\mathrm{Q.P_P}] + k_{pb}[\mathrm{QB.P_P}] \tag{2}$$

We assume that MgrB affects two rate constants. Autophosphorylation is suppressed by a factor $\lambda < 1$ whereas dephosphorylation is enchanced by a factor of $\gamma > 1$, so that $k_{apb} = \lambda k_{ap}$ and $k_{pb} = \gamma k_p$. Substituting complex concentrations from Equation 1:

$$\begin{aligned}
\frac{k_t}{K_{MT}}[\mathrm{Q_P}][\mathrm{P}] + \frac{k_{tb}}{K_{MTb}}[\mathrm{QB_P}][\mathrm{P}] &= \frac{k_p}{K_{MP}}[\mathrm{Q}][\mathrm{P_P}] + \frac{k_{pb}}{K_{MPb}}[\mathrm{QB}][\mathrm{P_P}] \\
\frac{k_t}{K_{MT}}\left([\mathrm{Q_P}][\mathrm{P}] + [\mathrm{QB_P}][\mathrm{P}]\right) &= \frac{k_p}{K_{MP}}\left([\mathrm{Q}][\mathrm{P_P}] + \gamma[\mathrm{QB}][\mathrm{P_P}]\right)
\end{aligned} \tag{3}$$

Steady state expressions for $[\mathrm{Q_P}]$ and $[\mathrm{QB_P}]$ can be found by first setting $\frac{d[\mathrm{Q_P}]}{dt}$ & $\frac{d[\mathrm{QB_P}]}{dt}$ to zero, followed by plugging in complex concentrations from 1. The expressions can then be plugged into Equation 2.

$$k_{ap}[\mathrm{Q}] = k_{-ap}[\mathrm{Q_P}] + k_t[\mathrm{Q_P.P}] \tag{4}$$

$$\lambda k_{ap}[\mathrm{QB}] = k_{-ap}[\mathrm{QB_P}] + k_t[\mathrm{QB_P.P}] \tag{5}$$

$$k_{ap}[\mathrm{Q}] = k_{-ap}[\mathrm{Q_P}] + \frac{k_t}{K_{MT}}[\mathrm{Q_P}][\mathrm{P}] \tag{6}$$

$$\lambda k_{ap}[\mathrm{QB}] = k_{-ap}[\mathrm{QB_P}] + \frac{k_{tb}}{K_{MTb}}[\mathrm{QB_P}][\mathrm{P}] \tag{7}$$

$$\left(\frac{C_t}{[\mathrm{P}]} + 1\right)([\mathrm{Q}] + \gamma[\mathrm{QB}]) = \frac{C_p}{[\mathrm{P_P}]}([\mathrm{Q}] + \lambda[\mathrm{QB}]) \tag{8}$$

The expression for [QB] in terms of [Q] and [B] can be found by setting $\frac{d[\mathrm{B}]}{dt}$ to zero.

$$[\mathrm{QB}] = \frac{[\mathrm{Q}][\mathrm{B}]}{K_D} \tag{9}$$

Plugging back into Equation 8,

$$\left(\frac{C_t}{[\mathrm{P}]} + 1\right)\left(1 + \gamma\frac{[\mathrm{B}]}{K_D}\right) = \frac{C_p}{[\mathrm{P_P}]}\left(1 + \lambda\frac{[\mathrm{B}]}{K_D}\right) \tag{10}$$

Where $C_t = \frac{k_{-ap}K_{MT}}{k_t}$ & $C_p = \frac{k_{ap}K_{MP}}{k_p}$.

This equation will be constrained by conservation equations for PhoP, PhoQ and MgrB. In general,

$$\begin{aligned}[\mathrm{P}]_T = {} & [\mathrm{P}] + [\mathrm{P_P}] + \\ & [\mathrm{QB.P_P}] + [\mathrm{QB_P.P}] + [\mathrm{Q_P.P}] + [\mathrm{Q.P_P}]\end{aligned}$$

However, two-component system histidine kinases are usually expressed at much lower concentrations compared to response regulators. Ratio of PhoP:PhoQ in *E. coli* is reported to be much larger than 1 [2, 3]. Therefore:

$$\begin{aligned}[\mathrm{P}]_T &\approx [\mathrm{P}] + [\mathrm{P_P}] \\ [\mathrm{B}]_T &= [\mathrm{B}] + [\mathrm{QB}] + [\mathrm{QB_P}] + [\mathrm{QB.P_P}] + [\mathrm{QB_P.P}] \\ [\mathrm{Q}]_T &= [\mathrm{Q}] + [\mathrm{Q_P}] + [\mathrm{Q_P.P}] + [\mathrm{Q.P_P}] \\ &\quad + [\mathrm{QB}] + [\mathrm{QB_P}] + [\mathrm{QB.P_P}] + [\mathrm{QB_P.P}]\end{aligned} \tag{11}$$

Negative transcriptional feedback dictates the following saturating relation between B-total and PhoP-P

$$[\mathrm{B}]_T = [\mathrm{B}]_T^0 + \frac{(f_B[\mathrm{B}]_T^0[\mathrm{P_P}]^2)}{(K_B^2 + [\mathrm{P_P}]^2)} \tag{12}$$

The set of equations 10,11,12 together form the complete model of PhoPQ TCS. If we set MgrB-total to zero, this set of equations reduces to the model in ref [1] and the concentration of [PhoP-P] is the negative solution of a quadratic as discussed in refs [1, 3, 4]. In general however, we solve equations 10,11,12 for [PhoP-P] assuming the following parameters (computed from the two-state models in Table 2, S5 Text): $C_p = 20\mu M, C_t = 2 \times 10^{-5}\mu M, K_D = 2 \times 10^{-3}\mu M, \lambda = 2.7 \times 10^{-4}, K_B = 0.2\mu M, f_B = 50$. For Figure 2B (Main Text), $[\mathrm{B}]_T^0 = 0.14\mu M$. $[\mathrm{B}]_T^0$ was varied from 0 to 5 (S1 Fig). At each $[\mathrm{B}]_T^0$, the equations were solved for a range of P-total values ($[\mathrm{Q}]_T = [\mathrm{P}]_T/40$) as shown in S1 Fig. Non-linear equations solved using fminbnd function in MATLAB to obtain steady state PhoP-P.

At high levels of $[\mathrm{B}]_T^0$, we can rewrite total MgrB conservation in equation 11 as $[\mathrm{B}]_T = [\mathrm{B}] + \epsilon$, where ϵ represents terms of the order $[\mathrm{Q}]_T/[\mathrm{B}]_T$. At the limit $\epsilon \to 0$, $[\mathrm{B}] \approx [\mathrm{B}]_T$. Equations 11 can then be incorporated in a straightforward way into eq 10.

$$\left(\frac{C_t}{[\mathrm{P}]_T - [\mathrm{P_P}]} + 1\right)\left(1 + \gamma\frac{[\mathrm{B}]_T}{K_D}\right) = \frac{C_p}{[\mathrm{P_P}]}\left(1 + \lambda\frac{[\mathrm{B}]_T}{K_D}\right) \tag{13}$$

As $[\mathrm{P}]_T$ increases, the term $C_t/[\mathrm{P}]$ (dependent on autodephosphorylation rate k_{-ap}) becomes less significant as explained previously in ref [1], leading to robustness of [PhoP-P] to total-PhoP,PhoQ. The limit $C_t \ll [P]_T$, which can be reformulated in terms of k_{-ap}, would ensure robustness away from saturation, i.e. when $[P_P]$ does not approach $[P]_T$. In this limit Eqs 10 and 13 become Eqs 2 and 3 in the main text respectively.

References

[1] E. Batchelor and M. Goulian. Robustness and the cycle of phosphorylation and dephosphorylation in a two-component regulatory system. *Proc Natl Acad Sci U S A*, 100(2):691–6, 2003.

[2] G. W. Li, D. Burkhardt, C. Gross, and J. S. Weissman. Quantifying absolute protein synthesis rates reveals principles underlying allocation of cellular resources. *Cell*, 157(3):624–35, 2014.

[3] T. Miyashiro and M. Goulian. High stimulus unmasks positive feedback in an autoregulated bacterial signaling circuit. *Proc Natl Acad Sci U S A*, 105(45):17457–62, 2008.

[4] G. Shinar, R. Milo, M. R. Martinez, and U. Alon. Input output robustness in simple bacterial signaling systems. *Proc Natl Acad Sci U S A*, 104(50):19931–5, 2007.
