# Supplementary material for: Overlaid positive and negative feedback loops shape dynamical properties of PhoPQ two-component system
Source: PLoS Comput Biol. 2021 Jan 4;17(1):e1008130. doi: 10.1371/journal.pcbi.1008130 (PMC7808668; doi:10.1371/journal.pcbi.1008130)
Supplement: S3 Text — (PDF) [file pcbi.1008130.s011.pdf]

S3 Text: Model predictions and analysis of oscillations

In order to understand the role of negative feedback in shaping the dynamical properties of the TCS, we constructed *in-silico* mutants of our wild-type models with negative feedback through *mgrB*-upregulation removed. We constructed *in-silico* mutants expressing *mgrB* constitutively. We find that models of wild-type PhoPQ TCS that fit well with temporal data as well as display biphasic dose-response can show limit cycle oscillations in PhoP-P if *mgrB* is expressed at a constant rate comparable to the basal *mgrB* expression rates of corresponding wild-type models, i.e. if *mgrB* upregulation by PhoP-P is removed from the wild-type model. We observe limit cycle oscillations at signal levels equivalent to ∼1 mM Mg^{2+}(Figure 1).

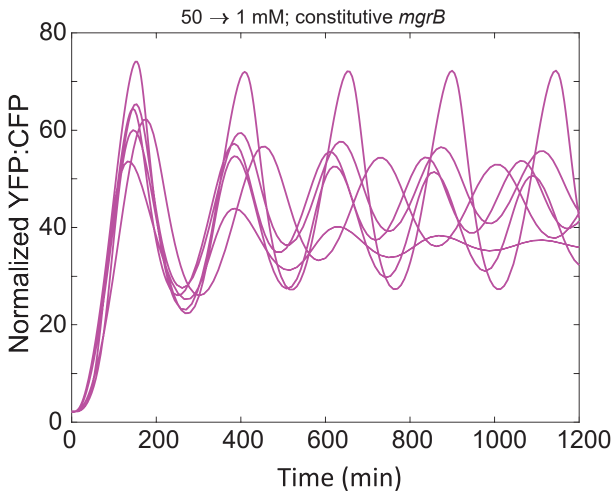

Figure 1: Following a switch in Mg^{2+}concentration from 50 to 1 mM, our PhoPQ models with constitutively expressed *mgrB* (at levels comparable to wild-type basal) predict oscillations in PhoP-P output. Each line represents a parameter set that explains wild-type data (including biphasic dose-response) as shown in S4 Fig

To understand the mechanism that results in limit cycle oscillations following removal of negative feedback through MgrB upregulation, we find a minimal model of the TCS that still retains oscillations (Figure 2). The minimal model consists of two conformations of the kinase (Q_{kin} and Q_{ph}). Each conformation catalyzes phosphorylation or dephosphorylation reactions with a Michaelis-Menten dependence on the substrate, P and P_P respectively. We

assume a constant amount of total PhoP that is much greater than PhoQ as positive autoregulation of PhoP doesn't contribute to the oscillations significantly. We find that positive feedback through upregulation of PhoQ (synthesized in a kinase-biased conformation Q_{kin}; Figure 2), together with a slow negative feedback through conversion of Q_{kin} to the phosphatase conformation (Q_{ph}) is sufficient to yield oscillations.

To check the effect of negative feedaback via upregulation of MgrB by PhoP-P in the wild-type modelin this toy model. We introduce increase in conformational switching rate K_f as a function of PhoP-P. With that model we find that feedback eliminates sustained oscillations by speeding up kinase to phosphatase switch.

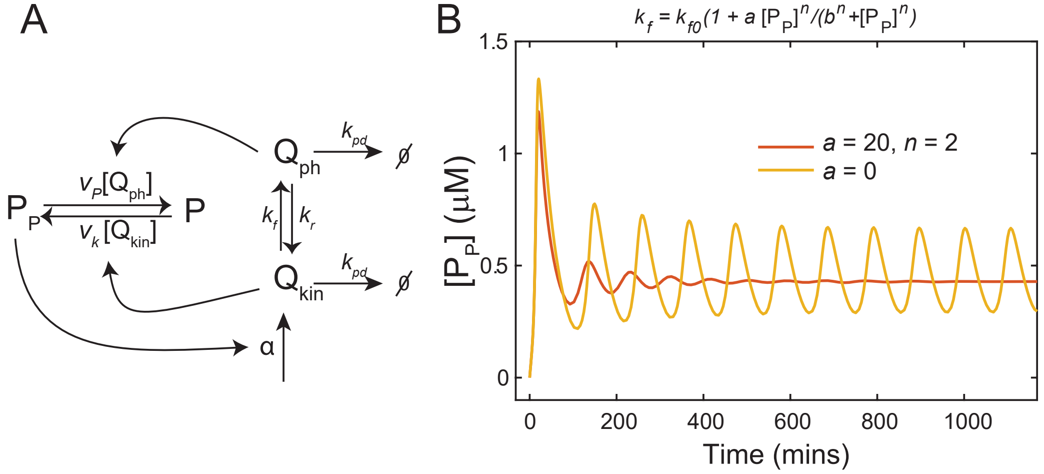

Figure 2: A - Minimal model of the model that yields oscillations in PhoP-P (P_P). Phosphorylation and dephosphorylation reactions are modeled as Michaelis-Menten reactions catalyzed by Q_{kin} and Q_{ph} respectively,i.e. $v_k = v_k^{max}[\mathrm{P}]/(K_k + [\mathrm{P}]), v_P = v_P^{max}[[\mathrm{P_P}]/(K_P + [\mathrm{P_P}])$. P_P drives synthesis of Q_{kin} with a saturating dependence $(\alpha = b\left(1 + f[\mathrm{P_P}]^2)/(K_0^2 + [\mathrm{P_P}])^2)\right))$. Q_{kin} undergoes a reversible conversion to Q_{ph}. The rates used in this model were derived from the parameters used in the fully descriptive two-state PhoPQ ODE model. B- The model yields limit cycle oscillations (yellow). If the rate of kinase to phosphatase transition is modeled to increase as a function of P_P (to recapitulate effects of MgrB upregulation), the oscillations are suppressed and result in a stable steady state (red). Rates (units: $\mu M, s$): $k_{pd} = 3.1 \times 10^{-4}, b = 1.2 \times 10^{-6}, f = 26, K_0 = 0.65, v_k^{max} = 3, K_k = 0.6, v_P^{max} = 1.5, K_P = 13.8, P_T = 1.6, k_f = 0.006, k_r = 1.5 \times 10^{-6}$
