# Supplementary material for: Overlaid positive and negative feedback loops shape dynamical properties of PhoPQ two-component system
Source: PLoS Comput Biol. 2021 Jan 4;17(1):e1008130. doi: 10.1371/journal.pcbi.1008130 (PMC7808668; doi:10.1371/journal.pcbi.1008130)
Supplement: S4 Text — (PDF) [file pcbi.1008130.s012.pdf]

S4 Text: A framework to examine steady state signal response for two-state model of PhoPQ-MgrB

Note on shorthand use in the figures and equations in all supplemental texts: We represent PhoQ by Q, MgrB by B and subscript P for phosphorylated state. For example, the complex PhoQ-P.MgrB is represented as QB_P.

In this section, we look at how this particular model (Fig 3 Main Text, Fig 2 in S5 Text, S6 Fig) that fits well to experimental data is able to simulate a biphasic dose-response. Therefore, the analysis here is not general but dependent on the particular parameter set resulting in simulations that match experimental data (See Fig 3 Main Text, S6 Fig). We make many assumptions along the way to attempt to find why PhoP-P can show a biphasic response to decreasing k_{-1}. As we show in Fig 4 (main text), the expressions we develop in this section for PhoP-P in ranges of k_{-1} corresponding to high ($>$ 1 mM) and intermediate (1-0.01mM) $Mg^{2+}$are good approximations of the steady state PhoP-P obtained from numerically solving the full model.

We analyze the model with no autoregulation, since that strain shows no difference in output compared to wild-type over the plateau region, both in experiments and in this model [1]. The mechanism for plateauing however remains valid even with autoregulation of *phoPQ*. Consider 4 forms of PhoQ: kinase, phosphatase and MgrB bound kinase, phosphatase. So the ODE

system for PhoQ concentrations reduces to:

$$
\begin{aligned}
\frac{d[\mathrm{Q_{kin}}]}{dt} &= \frac{d[\mathrm{Q}^*]}{dt} + \frac{d[\mathrm{Q_P}]}{dt} + \frac{d[\mathrm{Q_P.P}]}{dt} \\
&= k_{tlnQ}\mathrm{mPQ} + k_1[\mathrm{Q}] - k_{-1}[\mathrm{Q}^*] - k_b[\mathrm{Q}^*][\mathrm{B}] + k_d[\mathrm{Q}^*\mathrm{B}] - k_{pd}[\mathrm{Q_{kin}}] \\
\frac{d[\mathrm{QB_{kin}}]}{dt} &= \frac{d[\mathrm{Q}^*\mathrm{B}]}{dt} + \frac{d[\mathrm{QB_P}]}{dt} + \frac{d[\mathrm{QB_P.P}]}{dt} \\
&= k_b[\mathrm{Q}^*][\mathrm{B}] - k_d[\mathrm{Q}^*\mathrm{B}] + fk_1[\mathrm{QB}] - k_{-1}[\mathrm{Q}^*\mathrm{B}] - k_{pd}[\mathrm{QB_{kin}}] \\
\frac{d[\mathrm{Q_{ph}}]}{dt} &= \frac{d[\mathrm{Q}]}{dt} + \frac{d[\mathrm{Q.P_P}]}{dt} \\
&= k_{-1}[\mathrm{Q}^*] - k_1[\mathrm{Q}] - k_b[\mathrm{Q}][\mathrm{B}] + fk_d[\mathrm{QB}] - k_{pd}[\mathrm{Q_{ph}}] \\
\frac{d[\mathrm{QB_{ph}}]}{dt} &= \frac{d[\mathrm{QB}]}{dt} + \frac{d[\mathrm{QB.P_P}]}{dt} \\
&= k_b[\mathrm{Q}][\mathrm{B}] - fk_d[\mathrm{QB}] - fk_1[\mathrm{QB}] + k_{-1}[\mathrm{Q}^*\mathrm{B}] - k_{pd}[\mathrm{QB_{ph}}]
\end{aligned}
\tag{1}
$$

For simplicity, we do this analysis as $\lambda \to 0$ and $f \to 0$. We assume all catalytic conversions within each form happen much faster than conversions between them. Thus the instantaneous concentrations of all sub-forms depend on the parent form in the following way:

- Kinase:

$$
\begin{aligned}
[\mathrm{Q_{kin}}] &= [\mathrm{Q}^*] + [\mathrm{Q_P}] + [\mathrm{Q_P.P}] \\
[\mathrm{Q_P.P}] &= [\mathrm{Q_{kin}}]\frac{\frac{[\mathrm{P}]}{K_{MT}}}{1 + \left(1 + \frac{k_4}{k_2}\right)\frac{[\mathrm{P}]}{K_{MT}}} \\
[\mathrm{Q}^*] &= [\mathrm{Q_{kin}}]\frac{\left(\frac{k_4}{k_2}\right)\frac{[\mathrm{P}]}{K_{MT}}}{1 + \left(1 + \frac{k_4}{k_2}\right)\frac{[\mathrm{P}]}{K_{MT}}} \\
[\mathrm{Q_P}] &= [\mathrm{Q_{kin}}]\frac{1}{1 + \left(1 + \frac{k_4}{k_2}\right)\frac{[\mathrm{P}]}{K_{MT}}}
\end{aligned}
\tag{2}
$$

- Phosphatase:

$$\begin{aligned}[\mathrm{Q_{ph}}] &= [\mathrm{Q}] + [\mathrm{Q.P_P}] \\ [\mathrm{Q.P_P}] &= [\mathrm{Q_{ph}}]\frac{\frac{[\mathrm{P_P}]}{K_{MP}}}{1+\frac{[\mathrm{P_P}]}{K_{MP}}} \\ [\mathrm{Q}] &= [\mathrm{Q_{ph}}]\frac{1}{1+\frac{[\mathrm{P_P}]}{K_{MP}}}\end{aligned} \tag{3}$$

- MgrB-PhoQ Kinase:

$$\begin{aligned}[\mathrm{QB_{kin}}] &= [\mathrm{Q^*B}] + [\mathrm{QB_P}] + [\mathrm{QB_P.P}] \\ [\mathrm{QB_P.P}] &= 0 \\ [\mathrm{Q^*B}] &= [\mathrm{QB_{kin}}] \\ [\mathrm{QB_P}] &= 0\end{aligned} \tag{4}$$

- MgrB-PhoQ Phosphatase:

$$\begin{aligned}[\mathrm{QB_{ph}}] &= [\mathrm{QB}] + [\mathrm{QB.P_P}] \\ [\mathrm{QB.P_P}] &= [\mathrm{QB_{ph}}]\frac{\frac{[\mathrm{P_P}]}{K_{MP}}}{1+\frac{[\mathrm{P_P}]}{K_{MP}}} \\ [\mathrm{QB}] &= [\mathrm{QB_{ph}}]\frac{1}{1+\frac{[\mathrm{P_P}]}{K_{MP}}}\end{aligned} \tag{5}$$

Now, we look at phosphorylation and dephosphorylation fluxes of PhoP to compute steady state PhoP-P.

- Dephosphorylation of PhoP-P

  1. Phosphatase activity: Using paramters that generate optimal fit, we find that simulated $[\mathrm{P_P}]$ remains well below the Michaelis Menten constant K_{MP}. As a consequence, $[\mathrm{Q_{ph}}] \approx [\mathrm{Q}]$ & $[\mathrm{QB_{ph}}] \approx [\mathrm{QB}]$. Moreover, at all signal levels contribution of $\mathrm{Q_{ph}}$ is negligible compared to $\mathrm{QB_{ph}}$ owing to most Q molecules being in bound

(QB) state (Main text Fig 4E).

$$\begin{aligned}\text{Flux} &= k_6[\text{Q.P}_\text{P}] + k_6[\text{QB.P}_\text{P}] \\ &= k_6[\text{Q}_\text{ph}]\frac{\frac{[\text{P}_\text{P}]}{K_{MP}}}{1+\frac{[\text{P}_\text{P}]}{K_{MP}}} + k_6[\text{QB}_\text{ph}]\frac{\frac{[\text{P}_\text{P}]}{K_{MP}}}{1+\frac{[\text{P}_\text{P}]}{K_{MP}}} \\ &\approx k_6[\text{QB}_\text{ph}]\frac{[\text{P}_\text{P}]}{K_{MP}}\end{aligned} \tag{6}$$

2. Flux due to growth-dilution $= k_{pd}[\text{P}_\text{P}]$

- Phosphorylation of PhoP: We assume flux of autophosphorylation is almost equal to phosphotransfer i.e. auto-dephosphorylation flux ($k_{-2}[\text{Q}_\text{P}]$) is negligible. Thus, PhoP-P formation flux $\approx k_2[\text{Q}^*]$

At steady state,

$$k_2[\text{Q}^*] = k_6[\text{QB}_\text{ph}]\frac{[\text{P}_\text{P}]}{K_{MP}} + k_{pd}[\text{P}_\text{P}] \tag{7}$$

To compute $[\text{P}_\text{P}]$ at various signal levels (i.e. k_{-1} values) using this flux balance equation (Equation 7, we find approximate expressions for relation between $[\text{Q}^*]$ and $[\text{QB}_{ph}]$. To this end, the set of equations in 1 can be solved at steady state to yield:

$$k_b[\text{Q}^*][\text{B}]\frac{k_{-1}}{k_1 + k_b[\text{B}] + k_{pd}} + k_{-1}\frac{k_b[\text{Q}^*][\text{B}]}{k_d + k_{pd} + k_{-1}} = k_{pd}[\text{QB}_\text{ph}] \tag{8}$$

$[\text{B}]_T = [\text{B}] + \epsilon$, where ϵ represents total MgrB in PhoQ bound form. At all signal levels in our model, MgrB is in large excess of PhoQ, so that $\epsilon \sim 0$. As a consequence, MgrB binding PhoQ or PhoQ* is a pseudo first order reaction with a rate of $k_b[\text{B}]_T$.

High Mg^{2+}

At high Mg^{2+}levels, PhoQ* deactivation (to PhoQ) is much faster than PhoQ* binding MgrB i.e. $k_{-1} \gg k_b[\text{B}]$. Whereas at both high and intermediate Mg^{2+}levels deactivation rate is much larger than MgrB-PhoQ dissociation rate and growth-dilution rate i.e. $k_{-1} \gg k_d + k_{pd}$. Moreover, rate constant for PhoQ* or PhoQ binding MgrB is much larger than activation

rate of PhoQ to PhoQ* i.e. & $k_b[\mathrm{B}] \gg (k_1 + k_{pd})$. Therefore, the relation between [Q*] and [$\mathrm{QB_{ph}}$] i.e. Equation 8 reduces to:

$$k_{-1} \approx k_{pd}[\mathrm{QB_{ph}}]$$

Plugging back into the flux balance equation i.e. Equation 7,

$$\frac{k_2 k_{pd}}{k_{-1}}[\mathrm{QB_{ph}}] = k_6[\mathrm{QB_{ph}}]\frac{[\mathrm{P_P}]}{K_{MP}} + k_{pd}[\mathrm{P_P}]$$

$$\implies [\mathrm{P_P}] \approx \frac{\frac{k_2 k_{pd}}{k_{-1}}}{\frac{k_6}{K_{MP}} + \frac{k_{pd}}{[\mathrm{QB_{ph}}]}}$$

Since at this signal level $[\mathrm{QB_{ph}}] \approx [\mathrm{Q}]_T$, and nearly insensitive to signal (Main text Fig 4E), [$\mathrm{P_P}$] is inversely proportional to k_{-1}, i.e. increases with signal. This equation can be plotted for values of k_{-1} corresponding to high range of Magnesium concentrations (Main text Fig 4D, red dotted line).

Intermediate $\mathrm{Mg^{2+}}$

At intermediate $\mathrm{Mg^{2+}}$levels, relation between the pseudo-first order rate constant of PhoQ* binding MgrB and PhoQ* deactivation rate constant is the opposite of high $\mathrm{Mg^{2+}}$i.e. $k_{-1} \ll k_b[\mathrm{B}]$. Equation 8 can be approximated as:

$$k_{-1}\frac{k_b[\mathrm{Q^*}][\mathrm{B}]}{k_d + k_{pd} + k_{-1}} \approx k_{pd}[\mathrm{QB_{ph}}]$$
$$k_b[\mathrm{Q^*}][\mathrm{B}] \approx k_{pd}[\mathrm{QB_{ph}}]$$

Since

$$k_{-1} \gg k_d + k_{pd}, \frac{k_{-1}}{k_{-1} + k_d + k_{pd}} \approx 1$$

.

Plugging this relation between Q* and [$\mathrm{QB_{ph}}$] back into the flux balance equation 7, we get

$$\frac{k_2 k_{pd}}{k_b[\mathrm{B}]_T}[\mathrm{QB_{ph}}] = k_6[\mathrm{QB_{ph}}]\frac{[\mathrm{P_P}]}{K_{MP}} + k_{pd}[\mathrm{P_P}]$$
$$[\mathrm{P_P}] \approx \frac{\frac{k_2 k_{pd}}{k_b[\mathrm{B}]_T}}{\frac{k_6}{K_{MP}} + \frac{k_{pd}}{[\mathrm{QB_{ph}}]}} \tag{9}$$

Over this range,$[\mathrm{QB_{ph}}]$ can still be approximated as Q_T and considered independent of signal (Main text Fig 4E),

$$[\mathrm{P_P}] \approx \frac{\frac{k_2 k_{pd}}{k_b [\mathrm{B}]_T}}{\frac{k_6}{K_{MP}} + \frac{k_{pd}}{[\mathrm{Q}]_T}} \tag{10}$$

In this expression, there is no dependence on k_{-1}, but on total MgrB. Total MgrB is a function of $[\mathrm{P_P}]$, therefore steady state $[\mathrm{P_P}]$ remains nearly independent of k_{-1}(Main text Fig 4D). Promoter output plateaus over this range (Main text Fig 3). The plateau ceases to exist at lower $\mathrm{Mg^{2+}}$as $[\mathrm{QB_{ph}}]$ starts decreasing with signal (Main text Fig 4E), and $k_{-1} \sim k_d + k_{pd}$. The equation above can be solved for a value of $[\mathrm{P_P}]$ by solving the resulting cubic equation. Rearranging the above equation, $[\mathrm{P_P}][\mathrm{B}]_T = c$, where $c = \frac{\frac{k_2 k_{pd}}{k_b}}{\frac{k_6}{K_{MP}} + \frac{k_{pd}}{[\mathrm{Q}]_T}}$ is a constant independent of signal. $[\mathrm{B}]_T$ depends on $[\mathrm{P_P}]$ as noted in Equation 12 S2 Text. This results in a cubic in $[\mathrm{P_P}]$ which can be solved given the rate constants and value of $[\mathrm{Q}]_T$ to obtain $[\mathrm{P_P}]$ in this intermediate regime (black dashed line, Main text Fig 4D).

Taken together, this approximate analytical solution process allows us to pinpoint how specific parameter ranges coupled with the structure of the network creates regimes of dependence or independence of PhoP-P from signal levels.

References

[1] T. Miyashiro and M. Goulian. High stimulus unmasks positive feedback in an autoregulated bacterial signaling circuit. *Proc Natl Acad Sci U S A*, 105(45):17457–62, 2008.
