# Supplementary material for: Overlaid positive and negative feedback loops shape dynamical properties of PhoPQ two-component system
Source: PLoS Comput Biol. 2021 Jan 4;17(1):e1008130. doi: 10.1371/journal.pcbi.1008130 (PMC7808668; doi:10.1371/journal.pcbi.1008130)
Supplement: S5 Text — (PDF) [file pcbi.1008130.s013.pdf]

S5 Text: PhoPQ TCS models: Reactions, ODEs and Parameters

Model reactions: PhoPQ model with single bifunctional kinase

Note that for brevity in the figures and equations in all supplemental text files: We represent PhoQ by Q, MgrB by B and subscript P for phosphorylated state. For example, the complex PhoQ-P.MgrB is represented as QB_P.

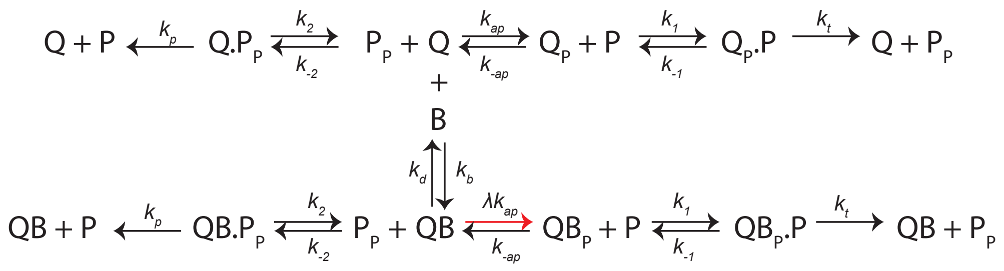

Figure 1: Figure shows all the reactions in PhoPQ TCS with associated rate constants. Reactions in the top row represent those in a typical TCS. This modified model includes Q binding B. QB undergoes all the same steps as Q (bottom row) with same rate constants, except autophosphorylation (shown by red arrow).

Post-translational reactions

- Signal sensing i.e. autophosphorylation; $k_{ap} = k^0_{ap} \frac{[\mathrm{Mg}^{2+}]^n}{K^n_{mg} + [\mathrm{Mg}^{2+}]^n}$

  $\mathrm{Q} \underset{k_{-ap}}{\overset{k_{ap}(\mathrm{signal})}{\rightleftharpoons}} \mathrm{Q_P}$

  $\mathrm{QB} \underset{k_{-ap}}{\overset{\lambda k_{ap}(\mathrm{signal})}{\rightleftharpoons}} \mathrm{QB_P}$

- PhoQ-MgrB binding

  $\mathrm{Q} + \mathrm{B} \underset{k_d}{\overset{k_b}{\rightleftharpoons}} \mathrm{QB}$

- Phosphotransfer

  $Q_P + P \underset{k_{-1}}{\overset{k_1}{\rightleftharpoons}} [Q_P \cdot P] \xrightarrow{k_t} Q + P_P$

  $QB_P + P \underset{k_{-1}}{\overset{k_1}{\rightleftharpoons}} [QB_P \cdot P] \xrightarrow{k_t} QB + P_P$

- Dephosphorylation

  $Q + P_P \underset{k_{-2}}{\overset{k_2}{\rightleftharpoons}} [Q \cdot P_P] \xrightarrow{k_p} Q + P$

  $QB + P_P \underset{k_{-2}}{\overset{k_2}{\rightleftharpoons}} [QB \cdot P_P] \xrightarrow{k_p} QB + P$

Transcription

Modeling of transcription and translation is the same for both models. PhoP-P binds promoter sites as a dimer, therefore we consider the following binding reactions. $P_{mgrB} + 2\,P_P \underset{k_{r1}}{\overset{k_{f1}}{\rightleftharpoons}} P_{mgrB} \cdot P_{P2}$

$P_{phoPQ} + 2\,P_P \underset{k_{r2}}{\overset{k_{f2}}{\rightleftharpoons}} P_{mgrB} \cdot P_{P2}$

We consider the binding reactions to be quasi-equilibrium state. Considering 1 copy of promoter per cell, the occupancy fraction of the promoter can be calculated as:

$$P_{mgrB}.P_{P2} = P_{mgrB}\frac{[P_P]^2}{K_1^2}$$

Here $K_1^2 = \frac{k_{r1}}{k_{f1}}$.

$$P_{mgrB} + P_{mgrB}.P_{P2} = 1$$

$$P_{mgrB}.P_{P2} = \frac{\frac{[P_P]^2}{K_1}}{1 + \frac{[P_P]^2}{K_1}}$$

Transcription from a promoter occurs at two rates: basal rate and active rate (when promoter is bound by the transcription factor). All transcripts degrade at a constant rate k_{md}

$P_{mgrB} \xrightarrow{k_{btpn1}} P_{mgrB} + m_{mgrB}$

$P_{mgrB} \cdot P_{P2} \xrightarrow{f_1 k_{btpn1}} P_{mgrB} + m_{mgrB}$

The same calculation gives a model for transcription for any mRNA (PhoP, PhoQ, MgrB or fluorescent reporter) from any PhoP-P dependent promoter (P_{mgrB} or P_{phoPQ}).

Translation

$\mathrm{m_{mgrB}} \xrightarrow{k_{tlnB}} \mathrm{m_{mgrB}} + \mathrm{B}$
$\mathrm{m_{phoPQ}} \xrightarrow{k_{tlnP}} \mathrm{m_{phoPQ}} + \mathrm{P}$
$\mathrm{m_{phoPQ}} \xrightarrow{k_{tlnQ}} \mathrm{m_{phoPQ}} + \mathrm{Q}$ Here k_{tlnQ} is assumed $\frac{1}{40}$ of k_{tlnP}.

Degradation

All proteins are assumed stable and dilute due to cell growth at a rate k_{pd}.

Model ODEs: PhoPQ model with single bifunctional kinase

$$
\begin{aligned}
\frac{d[\mathrm{P}]}{dt} &= k_{tlnP}m_{phoPQ} - k_1[\mathrm{Q_P}][\mathrm{P}] + k_{-1}[\mathrm{Q_P.P}] + k_p[\mathrm{Q.P_P}] \\
&\quad -k_1[\mathrm{QB_P}][\mathrm{P}] + k_{-1}[\mathrm{QB_P.P}] + k_p[QB.P_P] - k_{pd}[\mathrm{P}] \\
\frac{d[\mathrm{P_P}]}{dt} &= k_t[\mathrm{Q_P.P}] + k_t[\mathrm{QB_P.P}] - k_2[\mathrm{Q}][\mathrm{P_P}] + k_{-2}[\mathrm{Q.P_P}] \\
&\quad -k_2[\mathrm{QB}][\mathrm{P_P}] + k_{-2}[\mathrm{QB.P_P}] - k_{pd}[\mathrm{P_P}] \\
\frac{d[\mathrm{Q}]}{dt} &= k_{tlnQ}m_{phoPQ} - k_{ap}[\mathrm{Q}] + k_{-ap}[\mathrm{Q_P}] - k_b[\mathrm{B}][\mathrm{Q}] + k_d[\mathrm{QB}] \\
&\quad +k_t[\mathrm{Q_P.P}] - k_2[\mathrm{Q}][\mathrm{P_P}] \\
&\quad +k_{-2}[\mathrm{Q.P_P}] + k_p[\mathrm{Q.P_P}] - k_{pd}[\mathrm{Q}] \\
\frac{d[\mathrm{Q_P}]}{dt} &= k_{ap}[\mathrm{Q}] - k_{-ap}[\mathrm{Q_P}] + k_{-1}[\mathrm{Q_P.P}] - k_1[\mathrm{Q_P}][\mathrm{P}] - k_{pd}[\mathrm{Q_P}] \\
\frac{d[\mathrm{B}]}{dt} &= k_{tlnB}m_{mgrB} - k_b[\mathrm{B}][\mathrm{Q}] + k_d[\mathrm{QB}] - k_{pd}[\mathrm{B}] \\
\frac{d[\mathrm{QB}]}{dt} &= k_b[\mathrm{Q}][\mathrm{B}] - k_d[\mathrm{QB}] - \lambda k_{ap}[\mathrm{QB}] + k_{-ap}[\mathrm{QB_P}] + k_t[\mathrm{QB_P.P}] \\
&\quad -k_2[\mathrm{QB}][\mathrm{P_P}] + k_{-2}[\mathrm{QB.P_P}] + k_p[\mathrm{QB.P_P}] - k_{pd}[\mathrm{QB}] \\
\frac{d[\mathrm{QB_P}]}{dt} &= \lambda k_{ap}[\mathrm{QB}] - k_{-ap}[\mathrm{QB_P}] + k_{-1}[\mathrm{QB_P.P}] \\
&\quad -k_1[\mathrm{QB_P}][\mathrm{P}] - k_{pd}[\mathrm{QB_P}] \\
\frac{d[\mathrm{Q.P_P}]}{dt} &= k_2[\mathrm{Q}][\mathrm{P_P}] - k_{-2}[\mathrm{Q.P_P}] - k_p[\mathrm{Q.P_P}] - k_{pd}[\mathrm{Q.P_P}] \\
\frac{d[\mathrm{Q_P.P}]}{dt} &= k_1[\mathrm{Q_P}][\mathrm{P}] - k_{-1}[\mathrm{Q_P.P}] - k_t[\mathrm{Q_P.P}] - k_{pd}[\mathrm{Q_P.P}] \\
\frac{d[\mathrm{QB.P_P}]}{dt} &= k_2[\mathrm{QB}][\mathrm{P_P}] - k_{-2}[\mathrm{QB.P_P}] \\
&\quad -k_p[\mathrm{QB.P_P}] - k_{pd}[\mathrm{QB.P_P}] \\
\frac{d[\mathrm{QB_P.P}]}{dt} &= k_1[\mathrm{QB_P}][\mathrm{P}] - k_{-1}[\mathrm{QB_P.P}] \\
&\quad -k_t[\mathrm{QB_P.P}] - k_{pd}[\mathrm{QB_P.P}]
\end{aligned}
$$

$$
\begin{aligned}
\frac{d[m_{phoPQ}]}{dt} &= k_{btpn1}\frac{1+f_2\left(\frac{[\mathrm{P_P}]}{K_2}\right)^2}{1+\left(\frac{[\mathrm{P_P}]}{K_2}\right)^2} - k_{md}m_{phoPQ} \\
\frac{dm_{mgrB}}{dt} &= k_{btpn2}\frac{1+f_1\left(\frac{[\mathrm{P_P}]}{K_1}\right)^2}{1+\left(\frac{[\mathrm{P_P}]}{K_1}\right)^2} - k_{md}m_{mgrB} \\
\frac{d[m_{YFP2}]}{dt} &= k_{btpn2}\frac{1+f_2\left(\frac{[\mathrm{P_P}]}{K_1}\right)^2}{1+\left(\frac{[\mathrm{P_P}]}{K_1}\right)^2} - k_{md}[m_{YFP2}] \\
\frac{d[m_{YFP1}]}{dt} &= k_{btpn1}\frac{1+f_1\left(\frac{[\mathrm{P_P}]}{K_1}\right)^2}{1+\left(\frac{[\mathrm{P_P}]}{K_1}\right)^2} - k_{md}[m_{YFP2}] \\
\frac{d[YFP_1]}{dt} &= k_{tlnY}[m_{YFP1}] - k_{pd}[YFP_1] \\
\frac{d[YFP_2]}{dt} &= k_{tlnY}[m_{YFP2}] - k_{pd}[YFP_2]
\end{aligned}
$$

in-silico **mutants**

In-silico mutants are modeled in the same way for both dynamical models.

1. MgrB-deletion mutant, $\Delta mgrB$: Set $k_{btpn2} = 0$ in $\frac{d[m_{mgrB}]}{dt}$ only. This leads to zero MgrB but the reporter mRNA (mYFP2) and reporter protein (YFP) continue to report P_{mgrB} activity.

2. Autoregulation deletion: Set $f_1 = 1$ in $\frac{d[m_{phoPQ}]}{dt}$ only so that total PhoP, PhoQ remains constant but reporters mRNA (mYFP1) and protein (YFP1) levels continue to report P_{phoPQ} activity.

3. Constitutive *mgrB*: Set $f_2 = 1$ and vary k_{btpn2} in $\frac{d[m_{mgrB}]}{dt}$ only to set desired total MgrB expression at steady state, while mYFP2 and YFP2 continue to report on P_{mgrB} activity.

4. PhoQ-phosphatase activity mutant: Set all 3 rates associated with the Michaelis-Menten dephosphorylation reaction zero for Q and QB.

5. Double deletions will be combinations of individual *in-silico* mutants

Parameters: PhoPQ model with single bifunctional kinase

Parameter	Description	Units	Value

k_{pd}	Protein dilution rate	s^{-1}	3.1×10^{-4}
k_{md}	mRNA decay rate	s^{-1}	1.7×10^{-3}
k_{tlnP}	PhoP translation rate	s^{-1}	5.0×10^{-2}
k_{tlnB}	MgrB translation rate	s^{-1}	4.0×10^{-3}
k_{btpn1}	basal rate of transcription from P_{phoPQ}	mRNA copies s^{-1}	4.1×10^{-6}
k_{btpn2}	basal rate of transcription from P_{mgrB}	mRNA copies s^{-1}	8.7×10^{-6}
f_1	Fold increase in transcription rate from P_{phoPQ}	-	23
f_2	Fold increase in transcription rate from P_{mgrB}	-	55
K_1	Affinity of PhoP-P to P_{phoPQ}	μM	0.47
K_2	Affinity of PhoP-P to P_{mgrB}	μM	0.45
k_2	PhoQ autophosphorylation rate	s^{-1}	3.1
k_{-2}	PhoQ-P autodephosphorylation rate	s^{-1}	0
k_3	PhoQ-P to PhoP association rate	$\mu M^{-1} s^{-1}$	0.8
k_{-3}	[PhoQ-P.PhoP] dissociation rate	s^{-1}	1.2×10^{-2}
k_4	[PhoQ-P.PhoP] phosphotransfer rate	s^{-1}	2.1×10^{-1}
k_5	PhoQ to PhoP-P association rate	$\mu M^{-1} s^{-1}$	1.13
k_{-5}	[PhoQ.PhoP-P] dissociation rate	s^{-1}	9.2×10^{-4}
k_6	[PhoQ.PhoP-P] dephosphorylation rate	s^{-1}	2.16
k_{b1}	PhoQ to MgrB association rate	$\mu M^{-1} s^{-1}$	0.039
k_{d1}	[PhoQ.MgrB] dissociation rate	s^{-1}	5.6×10^{-4}

Table 1: Parameters used for one state PhoPQ model simulation (S4 Fig)

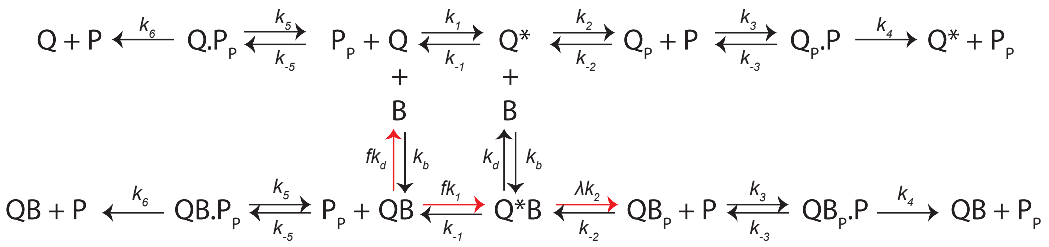

Figure 2: Figure shows all the reactions in the two-state PhoPQ TCS model with associated rate constants. Reactions in the top row represent those in a typical TCS. This modified model includes Q binding B. QB undergoes all the same steps as Q (bottom row) with same rate constants, except autophosphorylation and QB → QB* activation. For detailed balance, we assume QB dissociation is suppressed by the same ratio f. All suppressed rates denoted by red arrows.

Model reactions: PhoPQ model with two states of PhoQ

Post-translational reactions

- Signal sensing; $k_{-1} = k^0_{-1}[\text{Mg}^{2+}]$

  $\text{Q} \underset{k_{-1}\,\text{(signal)}}{\overset{k_1}{\rightleftharpoons}} \text{Q}^*$

  $\text{QB} \underset{k_{-1}\,\text{(signal)}}{\overset{k_{1b}}{\rightleftharpoons}} \text{Q}^*\text{B}$

- PhoQ-MgrB binding

  $\text{Q} + \text{B} \underset{k_d}{\overset{k_b}{\rightleftharpoons}} \text{QB}$

  $\text{Q}^* + \text{B} \underset{k_{d2}}{\overset{k_{b2}}{\rightleftharpoons}} \text{Q}^*\text{B}$

- Autophosphorylation

  $\text{Q}^* \underset{k_{-2}}{\overset{k_2}{\rightleftharpoons}} \text{Q}_\text{P}$

  $\text{Q}^*\text{B} \underset{k_{-2b}}{\overset{k_{2b}}{\rightleftharpoons}} \text{QB}_\text{P}$

- Phosphotransfer

$$\mathrm{Q_P + P \underset{k_{-3}}{\overset{k_3}{\rightleftharpoons}} [Q_P \cdot P] \xrightarrow{k_4} Q^* + P_P}$$

$$\mathrm{QB_P + P \underset{k_{-3b}}{\overset{k_{3b}}{\rightleftharpoons}} [QB_P \cdot P] \xrightarrow{k_{4b}} Q^*B + P_P}$$

- Dephosphorylation

$$\mathrm{Q + P_P \underset{k_{-5}}{\overset{k_5}{\rightleftharpoons}} [Q \cdot P_P] \xrightarrow{k_6} Q + P}$$

$$\mathrm{QB + P_P \underset{k_{-5b}}{\overset{k_{5b}}{\rightleftharpoons}} [QB \cdot P_P] \xrightarrow{k_{6b}} QB + P}$$

Detailed balance condition constrains parameters as follows:

$$\frac{k_{b1}}{k_{b2}} = \frac{k_{1b}}{k_1} \times \frac{k_{d1}}{k_{d2}}$$

Model ODEs: PhoPQ model with two states of PhoQ

$$\begin{aligned}
\frac{d[\mathrm{P}]}{dt} &= k_{tlnP}[m_{phoPQ}] - k_3[\mathrm{Q_P}][\mathrm{P}] + k_{-3}[\mathrm{Q_P.P}] \\
&\quad + k_6[\mathrm{Q.P_P}] - k_{3b}[\mathrm{QB_P}][\mathrm{P}] + k_{-3b}[\mathrm{QB_P.P}] \\
&\quad + k_{6b}[\mathrm{QB.P_P}] - k_{pd}[\mathrm{P}] \\
\frac{d[\mathrm{P_P}]}{dt} &= k_4[\mathrm{Q_P.P}] - k_5[\mathrm{Q}][\mathrm{P_P}] + k_{-5}[\mathrm{Q.P_P}] \\
&\quad + k_{4b}[\mathrm{QB_P.P}] - k_{5b}[\mathrm{QB}][\mathrm{P_P}] \\
&\quad + k_{-5b}[\mathrm{QB.P_P}] - k_{pd}[\mathrm{P_P}] \\
\frac{d[\mathrm{Q}]}{dt} &= -k_1[\mathrm{Q}] + k_{-1}[\mathrm{Q^*}] - k_{b1}[\mathrm{Q}][\mathrm{B}] + k_{d1}[\mathrm{QB}] - k_5[\mathrm{Q}][\mathrm{P_P}] \\
&\quad + k_{-5}[\mathrm{Q.P_P}] + k_6[\mathrm{Q.P_P}] - k_{pd}[\mathrm{Q}] \\
\frac{d[\mathrm{Q^*}]}{dt} &= k_{tlnQ}[m_{phoPQ}] + k_1[\mathrm{Q}] - k_{-1}[\mathrm{Q^*}] - k_2[\mathrm{Q^*}] + k_{-2}[\mathrm{Q_P}] \\
&\quad + k_4[\mathrm{Q_P.P}] - k_{b2}[\mathrm{Q^*}][\mathrm{B}] + k_{d2}[\mathrm{Q^*B}] - k_{pd}[\mathrm{Q^*}] \\
\frac{d[\mathrm{Q_P}]}{dt} &= k_2[\mathrm{Q^*}] - k_{-2}[\mathrm{Q_P}] + k_{-3}[\mathrm{Q_P.P}] - k_3[\mathrm{Q_P}][\mathrm{P}] - k_{pd}[\mathrm{Q_P}] \\
\frac{d[\mathrm{B}]}{dt} &= k_{tlnB}[m_{mgrB}] - k_{b2}[\mathrm{Q^*}][\mathrm{B}] + k_{d2}[\mathrm{Q^*B}] - k_{b1}[\mathrm{Q}][\mathrm{B}] + k_{d1}[\mathrm{QB}] - k_{pd}[\mathrm{B}] \\
\frac{d[\mathrm{QB}]}{dt} &= -k_{1b}[\mathrm{QB}] + k_{-1b}[\mathrm{Q^*B}] + k_{b1}[\mathrm{Q}][\mathrm{B}] - k_{d1}[\mathrm{QB}] - k_{5b}[\mathrm{QB}][\mathrm{P_P}] \\
&\quad + k_{-5b}[\mathrm{QB.P_P}] + k_{6b}[\mathrm{QB.P_P}] - k_{pd}[\mathrm{QB}] \\
\frac{d[\mathrm{Q^*B}]}{dt} &= +k_{1b}[\mathrm{QB}] - k_{-1b}[\mathrm{Q^*B}] - k_{2b}[\mathrm{Q^*B}] + k_{-2b}[\mathrm{QB_P}] + k_{4b}[\mathrm{QB_P.P}] \\
&\quad + k_{b2}[\mathrm{Q^*}][\mathrm{B}] - k_{d2}[\mathrm{Q^*B}] - k_{pd}[\mathrm{Q^*B}] \\
\frac{d[\mathrm{QB_P}]}{dt} &= k_{2b}[\mathrm{Q^*B}] - k_{-2b}[\mathrm{QB_P}] \\
&\quad + k_{-3b}[\mathrm{QB_P.P}] - k_{3b}[\mathrm{QB_P}][\mathrm{P}] - k_{pd}[\mathrm{QB_P}] \\
\frac{d[\mathrm{Q.P_P}]}{dt} &= k_5[\mathrm{Q}][\mathrm{P_P}] - k_{-5}[\mathrm{Q.P_P}] \\
&\quad - k_6[\mathrm{Q.P_P}] - k_{pd}[\mathrm{Q.P_P}]
\end{aligned}$$

$$
\begin{aligned}
\frac{d[\mathrm{QB.P_P}]}{dt} &= k_{5b}[\mathrm{QB}][\mathrm{P_P}] - k_{-5b}[\mathrm{QB.P_P}] \\
&-k_6 b[\mathrm{QB.P_P}] - k_{pd}[\mathrm{QB.P_P}] \\
\frac{d[\mathrm{Q_P.P}]}{dt} &= k_3[\mathrm{Q_P}][\mathrm{P}] - k_{-3}[\mathrm{Q_P.P}] \\
&-k_4[\mathrm{Q_P.P}] - k_{pd}[\mathrm{Q_P.P}] \\
\frac{d[\mathrm{QB_P.P}]}{dt} &= k_{3b}[\mathrm{QB_P}][\mathrm{P}] - k_{-3b}[\mathrm{QB_P.P}] \\
&-k_{4b}[\mathrm{QB_P.P}] - k_{pd}[\mathrm{QB_P.P}] \\
\frac{d[m_{phoPQ}]}{dt} &= k_{btpn1}\frac{1+f_2\left(\frac{[\mathrm{P_P}]}{K_2}\right)^2}{1+\left(\frac{[\mathrm{P_P}]}{K_2}\right)^2} - k_{md}m_{phoPQ} \\
\frac{dm_{mgrB}}{dt} &= k_{btpn2}\frac{1+f_1\left(\frac{[\mathrm{P_P}]}{K_1}\right)^2}{1+\left(\frac{[\mathrm{P_P}]}{K_1}\right)^2} - k_{md}m_{mgrB} \\
\frac{d[m_{YFP2}]}{dt} &= k_{btpn2}\frac{1+f_2\left(\frac{[\mathrm{P_P}]}{K_1}\right)^2}{1+\left(\frac{[\mathrm{P_P}]}{K_1}\right)^2} - k_{md}[m_{YFP2}] \\
\frac{d[m_{YFP1}]}{dt} &= k_{btpn1}\frac{1+f_1\left(\frac{[\mathrm{P_P}]}{K_1}\right)^2}{1+\left(\frac{[\mathrm{P_P}]}{K_1}\right)^2} - k_{md}[m_{YFP2}] \\
\frac{d[YFP_1]}{dt} &= k_{tlnY}[m_{YFP1}] - k_{pd}[YFP_1] \\
\frac{d[YFP_2]}{dt} &= k_{tlnY}[m_{YFP2}] - k_{pd}[YFP_2]
\end{aligned}
$$

Parameters: PhoPQ model with two states of PhoQ

Parameter	Description	Units	Value
k_{pd}	Protein dilution rate	s^{-1}	3.1×10^{-4}
k_{md}	mRNA decay rate	s^{-1}	4.1×10^{-3}
k_{tlnP}	PhoP translation rate	s^{-1}	3.7×10^{-2}
k_{tlnB}	MgrB translation rate	s^{-1}	2.3×10^{-2}
k_{btpn1}	basal rate of transcription from P_{phoPQ}	mRNA copies s^{-1}	4.97×10^{-6}
k_{btpn2}	basal rate of transcription from P_{mgrB}	mRNA copies s^{-1}	1.05×10^{-5}
f_1	Fold increase in transcription rate from P_{phoPQ}	-	26
f_2	Fold increase in transcription rate from P_{mgrB}	-	50
K_1	Affinity of PhoP-P to P_{phoPQ}	μM	0.62
K_2	Affinity of PhoP-P to P_{mgrB}	μM	0.19
k_1	PhoQ to PhoQ* activation rate	s^{-1}	3.2×10^{-3}

k_{-1}^0	PhoQ* to PhoQ deactivation rate (signal)	s^{-1}	8.7×10^{-2}
k_2	PhoQ* autophosphorylation rate	s^{-1}	12.5
k_{-2}	PhoQ-P autodephosphorylation rate	s^{-1}	0
k_3	PhoQ-P to PhoP association rate	$\mu M^{-1} s^{-1}$	5
k_{-3}	[PhoQ-P.PhoP] dissociation rate	s^{-1}	4.6×10^{-3}
k_4	[PhoQ-P.PhoP] phosphotransfer rate	s^{-1}	3.6×10^{0}
k_5	PhoQ to PhoP-P association rate	$\mu M^{-1} s^{-1}$	0.14
k_{-5}	[PhoQ.PhoP-P] dissociation rate	s^{-1}	3.6×10^{-3}
k_6	[PhoQ.PhoP-P] dephosphorylation rate	s^{-1}	1.56
k_{b2}	PhoQ to MgrB association rate	$\mu M^{-1} s^{-1}$	0.26
k_{d2}	[PhoQ.MgrB] dissociation rate	s^{-1}	1.0×10^{-4}

Table 2: Parameters used for two-state PhoPQ model (S6 Fig)
