# Supplementary material for: Overlaid positive and negative feedback loops shape dynamical properties of PhoPQ two-component system
Source: PLoS Comput Biol. 2021 Jan 4;17(1):e1008130. doi: 10.1371/journal.pcbi.1008130 (PMC7808668; doi:10.1371/journal.pcbi.1008130)
Supplement: Attachment — Submitted filename: PhoPQ_reviewerresponse_final.pdf [file pcbi.1008130.s014.pdf]

Dear Editor/Referees,

We are grateful for the overall positive assessment of the manuscript by the referees and the editor and very useful suggestions to improve the manuscript. I think most reviewers we in agreement that the paper provides a useful and previously unexplored angle on the biology pf PhoPQ systems and makes experimentally testable predictions. However, the reviewers were in unanimous agreement with respect to need to further demonstrations how our main conclusions depend on the assumptions about model parameters. Following these concerns, we have now included several supporting figures showing how our results hold in light of parameter perturbations. We have also revised the text to emphasize that our goal is to understand the underlying mechanism of the qualitative features of the steady state dose-response curve (lines 232-235). We further emphasize how our analytical approximations allow seeing parameter regimes in which these behaviors are possible (lines 232-235). Detailed responses are outlined below (in blue) and the text changes are highlighted in the manuscript in red.

Reviewer #1: This manuscript investigated how the negative feedback of MgrB regulation impacts the robustness of E. coli PhoPQ system, and explored potential mechanisms for MgrB feedback to produce the biphasic response. Two-component system is the prevalent bacterial signaling system that attracts extensive computational modeling. PhoPQ is one of the best studied two-component system in terms of robustness, response dynamics and feedback control. The results presented in this study would be of importance to the general understanding of the bacterial signaling mechanisms.

One of the major focus is to model the biphasic response. However, the main issue is that the extent of biphasic response has not been well characterized experimentally. I'm wondering how the modeling results depend on the actual quantitation of the biphasic response, particularly the highest activity relative to the intermediate plateau.

In Fig. 1C, the YFP:CFP output of the second phase is approximately five fold of the intermediate plateau. This appeared not consistent with the experimental data in the cited reference (Ref. 19). In Ref. 19 (Miyashiro et al 2008 PNAS), the intermediate plateaued output was ~0.6 shown in Fig.1C while the steady state output at growth-limiting Mg2+ was ~1 (Fig.3A in ref. 19), less than two fold of the plateau at 0.6 in Fig. 1C. Thus, for the biphasic data modeled in this study, there is uncertainty associated with both the x-scale (growth-limiting Mg2+ not well defined) and y-scale (fold difference to plateau). How will this impact the modeling?

-We thank the reviewer for highlighting the uncertainty in the highest activity relative to intermediate plateau. We have found a discrepancy in the data while coming up with an estimated maximum YFP:CFP value of the second phase relative to the intermediate plateau. We agree that an intermediate plateau value of ~0.6 (at 1 mM) and a maximum (at growth-limiting Mg2+) of ~ 1 (Figure 3A, Miyashiro and Goulian, PNAS 2008) yields an estimate of fold

change less than two fold. However, the initial YFP:CFP level in Fig 3A is < 0.2. The experiment in Fig 3A was conducted by growing cells overnight in 1mM Mg2+ followed by a thousand-fold dilution into fresh medium containing no added Mg2+. We contend that YFP:CFP at t = 0 in Fig 3A should then represent the steady state value of YFP:CFP at 1mM Mg2+ if no significant consumption of Mg2+ is assumed. This puts it at odds with the value of 0.6 in Fig 1C (Miyashiro and Goulian 2008). Due to this discrepancy, we concluded that comparing YFP:CFP values across figures with some differences in protocol may not be accurate.

Therefore, we decided to use the value on Figure 3A to estimate fold change range of 5-10 between intermediate plateau level and highest activity level (1/~0.2). We realize this is not a concrete estimate, and that better measurements can improve uncertainty associated with the y-axis. We believe that the existence of a biphasic response is the central question, and the highest activity relative to plateau activity does not play a major role in our study. To demonstrate this, we run our parameter fitting again, this time with a ratio of 2 (or more) between maximal and plateau activity of PmgrB. We find that the model is able to show a biphasic dose-response and fit temporal data as well (S7 Fig). Thus, the uncertainty in the y-axis does not alter our modeling conclusions and we update our text to mention this (lines 211-212). We believe that uncertainty on the x-axis (unknown growth-limiting Mg2+ concentration) is less impactful to our study. Considering half-maximal growth concentration of Mg2+ is reportedly 1-3 μM (Lusk et al 1968), and that the initial concentration in Fig 3A is definitely less than or equal to 1 μM (thousand-fold dilution of a ~1 mM Mg2+ medium), we assume 0.1 μM to be a good estimate of growth limiting Mg2+.

The following are my other comments:

1. Fig. 2, what is the unit of PhoPT concentration? Cp+Ct? It is better to state the unit in the figure legends. Can the modeling results be put into context of the actual measured cellular concentrations of PhoP?

Both [PhoP]T and [PhoP-P] were indeed plotted in (Cp + Ct) units to illustrate the generic nature of the phenomena. However, in light of the reviewer's suggestion and the concern of reviewer #3's regarding the robustness analysis shown in Figure 2, we now updated the figure. Instead of assumed parameters (previous S2 Text, page 3) we compute parameters equivalent to the two-state model and plot concentrations in non-normalized units (μM). To infer how the axis scale compares with wild-type levels of the proteins, we were able to find absolute synthesis rates of PhoP and PhoQ in Li et. al 2014 Cell in rich medium. The synthesis rates were reported to be 1508 and 130 molecules per generation, respectively. Assuming both proteins are stable, and depletion is due to growth-dilution, this translates to cellular concentrations of ~ 2.5 μM and 0.2 μM for PhoP and PhoQ respectively. The concentration of Mg2+ in this medium has been reported as 0.528 mM (Neidhardt, 1974). We have added a dashed black line to Figure 2B to indicate measured cellular concentration of PhoP demonstrating the systems operating in the robust regime.

2. I understand that the value of PhoP:PhoQ ratio will likely have minimal effect on the modeling conclusion. But I'm curious why a ratio of 40 was used instead of the actual measured value (7-18, Yeo et al Mol. Cell 2012; ~19, Li et al Cell 2014).

Miyashiro and Goulian 2008 PNAS reported a PhoP:PhoQ ratio of 50 (data not provided) and Li et al Cell 2014 reported a value of ~20. Yeo et al 2012 report the concentration ratio of PhoP:PhoQ in Salmonella. Given that we focused our analysis on questions specific to E. coli measurements, we used a value of 40. We now include a supporting figure showing that our conclusions are robust to modest variations of PhoP:PhoQ in the 20-50x range (Fig S2B). Lines 148:149

ACR theorem analysis shows that for irreversible autodephosphorylation of PhoQ robustness holds regardless of the relative ratio. In our numerical work in the presence of MgrB, robustness of the PhoPQ model does not greatly depend on the ratio (Fig S2B)

3. The impact of MgrB feedback on robustness clearly depends on the inhibition factor λ and MgrB-PhoQ affinity. Is it possible to comment how the values were chosen for the two parameters and how would they affect robustness?
The analytical solution for [PhoP-P] derived in S1 Text shows that robustness depends on MgrB-total being in large excess of PhoQ. At a given signal level, values of λ, KD set the steady state value of [PhoP-P] in the robust range of PhoPQ-total expression. We show that with 10-fold increase in λ (Fig S2A, dashed lines) or 10-fold increase in KD (Fig S2A, dotted lines) the range and the value of PhoP~P plateau may change but the existence of the plateau over more than 20-fold change in PhoP expression level is still observed. We include this control in supporting Fig S2. (Lines 148:149)
4. Fig. 3, please label the reactions (blue arrows) with the constant k-1, this may help readers to better follow the texts.
We labelled the reaction arrows in Fig 3.

5. I think the two-state model recapitulates accurately the conformation switching mechanism of histidine kinases. Can this model be used to explore robustness?

We agree that there is an added value in revisiting the robustness question for the developed two-state model. Following the referee's suggestion, we analyzed response of the two-state model for robustness to PhoPQ overexpression. The model shows a range in which variations of PhoP expression does not lead to significant changes in PhoP-P (Fig S8B). We also demonstrate that the two-state model can fit the experimentally measured data on the response of PmgrB promoter to overexpression of phoPQ operon from Miyashiro and Goulian 2008 (Fig S8A). (Lines 220-225)

6. Line 291-293, it was predicted that the time-delay between positive and negative feedbacks gives rise to oscillations. The negative feedback here is the conformation switching from a kinase to a phosphatase. It is hard to imagine that the timescale of conformational switching

would be significantly slower than the kinase reaction and protein expression. Thus oscillation appears very unlikely for mechanism alone.

Slow timescale of conformational switching naturally came out of the parameter fits that aimed to explain the biphasic dose response curve. Note that we hypothesize that the kinase to phosphatase switching rate decreases with Mg2+ decrease, therefore the switching timescale is dependent on signal level. Indeed, if the switching were fast, some of the analysis in S4 Text would not hold. For example, at intermediate Mg2+, where we predict oscillations in the absence of MgrB upregulation, we find that synthesized PhoQ kinase molecules dominantly bind MgrB, i.e $k_b[B] >> k_{-1}$. This condition is key to our explanation of why PhoP-P remains insensitive to increase in k_{-1}, i.e. key for explaining biphasic dose at intermediate Mg2+ as shown in S4 Text. Therefore, we think the slow switching (at intermediate Mg2+ where oscillations are seen) is a prediction of the model. We now pinpoint this in the manuscript (lines 307-310). Furthermore, given that the effective switching rate is greatly affected by the third protein MgrB and that the switching may be accompanied by the cofactor exchange, e.g. ATP/ADP we do not find this prediction to be very surprising. Perhaps experimental tests of the oscillations or direct measurements of the switching dynamics can be used to test these predictions in the future.

7. Line 332, PmgrB is a weaker promoter than PphoPQ? So, there is a possibility that MgrB becomes no longer in excess to PhoQ when PhoPQ is fully activated at growth-limiting Mg2+?

This is in fact a typo that is now corrected. We have corrected our manuscript to say “PmgrB is a stronger promoter than PphoPQ” (line 350). From Li et. al 2014 measurements in rich (MOPS complete) medium, we infer PhoQ and MgrB concentrations to be ~.2 and 1.5 μM respectively (at Mg2+ = 0.528 mM). The parity of this relationship is unlikely to change with decreasing Mg2+ due to stronger fold-change in the PmgrB promoter (estimated max fold activation of ~60 (Ram and Goulian, 2013, PloS Genetics), compared to ~20 that can be computed from YFP measurements from PphoPQ-yfp in Salazar et al 2016). We now discuss this point in the main text. (Lines 351-357)

Reviewer #2: The paper provides, via a mathematical model based on ODEs, a mechanistic explanation for the dynamical behaviour observed in PhoPQ two-component system. The models describe the interactions of the TCS with a negative feedback mechanism that is also regulated by the TCS response regulator. The model mechanisms are justified mainly by references to the papers 16,17,18,19. The results elucidate the effect of negative feedback on the TCS activity, in particular, the biphasic steady state response and suppression of oscillations.

The authors make simplifying assumptions, however they give careful explanations for the results. A strength of the paper is that the models are calibrated by experimental data from the references 16 and 19.

My main concern about the paper is the lack of an analysis of the parameter space: the claims

are rather on the structural aspects of the system although the model is parameter-rich with about 20-22 rate constants, listed in Tables 1 and 2 in S1, and some other constants for the boundary conditions... Some of the choices for these values are somewhat justified in terms of fitting to experimental data and other assumptions. However, it is not clear how much the results rely on the rate constants and how much they are due to structural properties of the system, e.g., inhibition by MgrB or the two states of PhoQ. A sensitivity analysis, for example, would help to address this issue.

My feeling is that such a model could be fit to produce a variety of dynamic behaviours, including the ones in the paper. In my opinion the authors need to address why the presented models are more adequate in comparison to any other, which might again fit to experimental data with the availability of a large parameter space.

-The goal of the paper was to provide an explanation to two qualitative features of the steady-state dose-response curves of the model – robustness of steady state to PhoPQ overexpression in the presence of negative feedback and existence of biphasic response curve. Never in the manuscript do we claim that either of these properties result from purely the "structural aspects" of the network. In fact, we attempt to construct simplified analytical models to precisely point out what parametric regimes enable the observed behaviors. For instance, such analysis pinpoints that MgrB should be in access to PhoQ (the observation confirmed by the data). As we now demonstrate numerically, some details of MgrB-PhoQ interactions can affect the exact shape of the dose-response curve but the plateau robustly shows up. Arguably, analytical model explaining why it is the case are more informative than numerical sensitivity analysis for all the parameters of the model.

Same arguments can be brought up with regards to biphasic response model. This behavior is not too robust and only small fraction of the parameter values are able to match the observed behavior. However, our goal was to ask if the biphasic response can arise without specific previously hypothesized mechanisms (multi-step activation via Mg2+binding/dissociation at multiple sites with different affinities) and if yes, illustrate the reason for it. The combination of numerical and analytical approaches presented fulfil that goal.

We note that most biologically meaningful predictions of the paper rely on the failures of a model to find any parameter set rather than on fit successes. We demonstrate how the network architecture fail to produce biphasic response unless certain "structural aspects" of the model are included. For instance, only in the model with two-states PhoQ (phosphatase and kinase) and with MgrB modulating transition between these and autophosphorylation rates are able to match the available data. We hope such predictions will be tested in the future experimental work.

Undoubtedly additional time-course measurements are required to fully constrain model parameters. Predicted oscillations (sustained or damped) or lack of thereof can provide a strategy to further refine the model with additional experimental measurements.

There are punctuation typos in the abstract, as well as the following:
positive feedback includes -> positive feedback, includes
Noun articles are missing as well.

There are grammatical mistakes, e.g., affect networks -> affects the network's
-We have corrected the text according to the suggestions above and proofread the paper.

The following statement in the introduction is not accurate: "In absence of environmental stimuli, sensor kinases sometimes have phosphatase activity, i.e. they can dephosphorylate the response regulator." These TCS respond to starvation, so it is a kind of a reverse mechanism. This might require highlighting.
Following this suggestion we explicitly mention Mg2+ replete conditions as "the absence of stimuli" conditions. (Line 20)

There is something wrong with the sentence in the "author summary": "our results show the advantages of such a combination feedback loops and predict the effect of their perturbation on the steady state and dynamic responses."
We thank the reviewer for bringing this to our attention and improve the author summary for clarity. (lines 9-11)

Reviewer #3: I found this to be an elegant and very interesting modeling study. The work is motivated by two concrete questions concerning the E. coli PhoQ/PhoP system—the fate of robustness when negative feedback on the kinase occurs and also the existence of biphasic behavior in dose-response curved. The authors come up with clear conclusions regarding both through an elegant combination of analytical and numerical studies. They also make a number of other interesting observations that follow from their two-state model. As the authors note, much if not most of their analysis is likely to be important for many other two-component systems. It is also notable that many predictions from this work can be tested experimentally.
-We thank the reviewer for the kind assessment.
I have a few comments that the authors should address.

1) lines 38-39 "PhoP activates transcription of a small lipoprotein (SlyB in 38 Salmonella and Yersinia pestis, MgrB in E. coli) that limits kinase activity" This sentence suggests that MgrB and SlyB are the same protein with different names in different organisms, or that SlyB is only in Salmonella and Yersina and MgrB is only in E.coli. However, they are different proteins and both are found in all three organisms. (MgrB was shown to affect PhoQ-regulated gene expression in all three organisms in ref 17.) Also, SlyB is a lipoprotein whereas MgrB is an integral membrane protein. The sentence should therefore be reworded.
We updated the text to accurately reflect the role of MgrB. Lines 38-40.

2) Along, the same lines as above, lines 370-371 and lines 355-356 seem to imply that Yersina and Salmonella do not have MgrB and instead have other proteins that limit PhoQ activity. These sentences should therefore be re-worded. Also, the authors may want to mention Klebsiella since MgrB inactivation in this bacterium has received a great deal of attention due to its role in colistin resistance.
We thank the reviewer for bringing the role of MgrB in colistin resistance in Klebsiella to our attention. We include it in our discussion. Lines 375-380.

3) lines 99-100 and 104-108. The claim about ACR confuses me. In the Supplement S1 text, the reactions labeled A describe a model that looks to be the same as the model in reference 20 for EnvZ-OmpR. But in reference 20, it is shown that the model only leads to approximate robustness. In References 21,22, on the other hand, ACR is demonstrated for EnvZ-OmpR in the limit in which the reverse reaction from EnvZ-P to EnvZ is neglected . Are the authors claiming that the full model, with the reverse reaction rate k_{-ap} not set to zero, has ACR, and therefore the claim of approximate robustness of the model in reference 20 is incorrect? If so, then this needs to be explained in detail. If not, then the discussion of ACR should be revised.

We are grateful to the reviewer for this comment that pinpointed a mistake in supporting text/figure discussing ACR analysis. Indeed, only when autophosphorylation of PhoQ is irreversible, conditions of Shinar- Feinberg theorem hold and robustness is expected. We now expand that discussion and color-code nodes in the network according to their “terminal”/”non-terminal” status. The supporting text (S1 Text) now discusses that when the phosphorylation reaction is reversible, node Q is terminal and the theorem is not applicable. (Lines 100-101)

4) There is relatively little discussion about K_D, the dissociation constant of MgrB-PhoQ. For the analysis of robustness, K_D was 0.1 in units of (Cp+Ct) (S2 text, p.3). How important is the magnitude of K_D for the approximate robustness? It would be helpful to include a few sentences discussing this.

K_D value doesn’t seem to be important to the robustness existence in the one-state PhoPQ model. Instead it, along with λ, sets the value of [PhoP-P] in the robustness regime. We have added a supporting figure to illustrate the importance of K_D for approximate robustness (Fig S2A). (Lines 148-149).

5) For the two-state model used to explain biphasic behavior, the association and dissociation rates k2b and k2d in Table 2, p. 9 of S1 Appendix, look like they give a MgrB-PhoQ dissociation constant that is about 4000-fold lower than the value of used for the robustness analysis (0.1 vs ~ 4*10^(-4)). Is there still a reasonable range of approximate robustness in this case for this much lower K_D? Also, it looks like there is a typo in the table - I assume these constants should be k2b and k2d not k1b and k1d.

-There is indeed a typo and we have corrected it in the S1 Appendix (now S5 Text). Furthermore, to ensure that the parameters used in the robustness analysis were not significantly different from those used in the two-state model, we now use the parameters for the one-state model that are computed from equivalent parameters of the two-state model. With these updated parameters we still observe similar approximate robustness and we update Figure 2B in the main text.

6) If, in the two state model, MgrB lowers the rate of the PhoQ -> PhoQ* transition, then wouldn't this mean that for the model used to analyze robustness, MgrB should increase the phosphatase rate in addition to lowering the autophosphorylation rate. It seems to me that in

an in vitro experiment one would see significantly faster dephosphorylation if high levels of MgrB are present. As far as I know, it is still an open question whether MgrB affects the phosphatase rate, so this is not necessarily a problem, but the authors should at least comment on this.

We thank the reviewer for raising this effect of two-state model on the prior analysis of robustness. Indeed, our analysis can be easily generalized to consider this effect and we now include this in the modified manuscript. When we use a factor $\gamma > 1$ for the increased phosphatase rate of [PhoQ.MgrB] in the analytical solution (S2 Text) would result in new form of the equation (2) main text

$$[PhoP-P] \approx \frac{C_p\left(1+\frac{\lambda[MgrB]}{K_D}\right)}{1+\frac{\gamma[MgrB]}{K_D}}$$

Despite this effect, the robustness analysis remains unchanged.

Furthermore, we agree that our modeling does indicate that higher dephosphorylation rates may be observed in vitro with higher MgrB levels added since MgrB slows the kinase form transitions. It remains to be seen whether this effect is observable *in vitro* given the already fast time scales of PhoQ phosphatase activity, or *in vivo* given the already high abundance of MgrB compared to PhoQ.
